# Identification and Phytotoxicity Assessment of Phenolic Compounds in *Chrysanthemoides monilifera* subsp. *monilifera* (Boneseed)

**DOI:** 10.1371/journal.pone.0139992

**Published:** 2015-10-14

**Authors:** Md Abdullah Yousuf Al Harun, Joshua Johnson, Md Nazim Uddin, Randall W. Robinson

**Affiliations:** Institute for Sustainability and Innovation, College of Engineering and Science, Victoria University, Melbourne, Australia; University of A Coruña, SPAIN

## Abstract

*Chrysanthemoides monilifera* subsp. *monilifera* (boneseed), a weed of national significance in Australia, threatens indigenous species and crop production through allelopathy. We aimed to identify phenolic compounds produced by boneseed and to assess their phytotoxicity on native species. Phenolic compounds in water and methanol extracts, and in decomposed litter-mediated soil leachate were identified using HPLC, and phytotoxicity of identified phenolics was assessed (repeatedly) through a standard germination bioassay on native *Isotoma axillaris*. The impact of boneseed litter on native *Xerochrysum bracteatum* was evaluated using field soil in a greenhouse. Collectively, we found the highest quantity of phenolic compounds in boneseed litter followed by leaf, root and stem. Quantity varied with extraction media. The rank of phenolics concentration in boneseed was in the order of ferulic acid > phloridzin > catechin > p-coumaric acid and they inhibited germination of *I*. *axillaris* with the rank of ferulic acid > catechin > phloridzin > p-coumaric acid. Synergistic effects were more severe compared to individual phenolics. The litter-mediated soil leachate (collected after15 days) exhibited strong phytotoxicity to *I*. *axillaris* despite the level of phenolic compounds in the decomposed leachate being decreased significantly compared with their initial level. This suggests the presence of other unidentified allelochemicals that individually or synergistically contributed to the phytotoxicity. Further, the dose response phytotoxic impacts exhibited by the boneseed litter-mediated soil to native *X*. *bracteatum* in a more naturalistic greenhouse experiment might ensure the potential allelopathy of other chemical compounds in the boneseed invasion. The reduction of leaf relative water content and chlorophyll level in *X*. *bracteatum* suggest possible mechanisms underpinning plant growth inhibition caused by boneseed litter allelopathy. The presence of a substantial quantity of free proline in the target species also suggests that the plant was in a stressed condition due to litter allelopathy. These findings are important for better understanding the invasive potential of boneseed and in devising control strategies.

## Introduction

Globally, the invasion of exotic species is one of the most important challenges experienced by native ecosystems. It is generally accepted that successful invasion by exotic species is determined by the characteristics of the invaded habitat [1] and biological attributes of the invader, including allelopathy [2, 3]. The novel weapon hypothesis suggests that allelopathy is one of the most influential mechanisms that permits plants to invade and establish in new ecosystems and which ultimately determines the structure and composition of the invaded plant community [4, 5]. The elucidation of plant-plant allelopathic interactions as distinct from resource competition has been challenged [6] and requires sufficient evidence by the authors [7, 8]. Among the identified allelochemicals, phenolic compounds are a class of the most important. They are ubiquitous in all plant organs that have been the subject of chemical, biological, agricultural, and medical studies in last few decades [9–12]. Despite evidence revealing that phytotoxicity depends on the types and quantity of allelochemicals released by the donor plants [12], there is still lack of information about the presence of specific allelochemicals in many invasive species. Not all allelochemicals contained and released by the allelopathic plants are phytotoxic to associated species and indeed, phytotoxicity may vary depending on dosage or concentration [12, 13]. The identification and quantification of specific allelochemicals is imperative in characterising the basis of allelopathy in plants and may also serve as a source of novel, natural herbicides, pesticides and medicines.

Australia is facing challenges in controlling boneseed (*Chrysanthemoides monilifera* subsp. *monilifera*) [14, 15]. Boneseed was introduced from South Africa in the mid-nineteenth century [15] and was proclaimed a noxious weed in Victoria in 1969 [16]. It is now a Weed of National Significance (WoNS) in Australia and is listed on the National Pest Plant Accord in New Zealand. Another subspecies of *Chrysanthemoides monilifera* found in Australia is subsp. *rotundata* (bitou bush) which is also an important weed in Australia. Boneseed and bitou bush collectively threaten about 200 indigenous species in Australia [17] including significant rare species such as *Pterostylis truncata*. McAlpine *et al*. [18] postulated that boneseed posed a severe threat to native species regeneration. Previous work [19] examining the suppression of native species in Australia by bitou bush through the release of allelochemicals led to the suspicion that boneseed may invade and threaten native species through the same mechanism. The previous study on mechanism of boneseed invasion [20] confirmed the phytotoxicity of boneseed but did not elucidate the role of allelopathy clearly due to the extraction procedure of chemical compounds. Therefore, allelopathy of this species during its invasive process is still to be demonstrated.

Of the potentially allelopathic compounds, present in invasive species phenolic compounds, in particular polyphenolics are the most commonly identified. Dietary role [21], medicinal importance [22], herbicidal potential [23] and phytotoxicity [24] of polyphenolics have been established earlier. Polyphenolic compounds like catechin, ferulic acid, etc. have been found to be substantially phytotoxic [12, 25]. A group of allelochemicals including phenolics have previously been identified in bitou bush although the identification and quantification of specific phenolic compounds was not investigated, despite their importance. Our previous study [20] identified high concentrations of total phenolics in boneseed compared with other allelopathic plant species [26]. As a part of chemo-ecological investigation of boneseed, and in an extension of the study by Ens *et al*. [19] on bitou bush, we aimed to identify individual phenolic compounds present in boneseed.

Phytotoxicity assessments of allelopathic species are mostly limited to bioassay experiments with aqueous extracts. Comparatively few of these studies have conducted phytotoxicity experiments based on the concentration of specific allelochemicals present in allelopathic species [27], despite the fact that these would more closely mimic the effects of these chemicals in the native environment. As a part of our overall investigation into the mechanisms of boneseed invasion, we previously studied the allelopathic potential of boneseed aqueous extract and leachate [20], and volatile chemicals and root exudates (unpublished) in terms of biochemical and physiological impacts on experimental native and model species. Test species from the same or similar habitat where allelopathic species grow may more authentically reveal the phytotoxic potential of the invasive species. To this end, *I*. *axillaris and X*. *bracteatum*, which grow in the same environment as boneseed and have previously been identified as susceptible to boneseed phytotoxicity [20] represent good test species for the allelopathic impact of boneseed.

Laboratory bioassay experiments based solely on aqueous extracts and standard allelochemicals in the extracts may not fully elucidate true phytotoxic effects. Field studies are imperative to demonstrate more clearly allelopathic impacts because edaphic and environmental factors work together to influence allelopathic effects [28]. Previous studies [29, 30] addressing the breakdown of soil allelochemicals by microorganisms and the consequent reduction of phytotoxicity reinforce the importance of allelopathic phytotoxicity study in soil systems. Boneseed sheds allelochemical-laden litter throughout the year although the quantity varies with season and geographical location [31, 32]. Our previous study revealed substantial degradation of total phenolics level in leachate of boneseed litter-mediated soil over time [33]. However, the identification of individual phenolic compounds and demarcation of phytotoxicity of natural leachate have yet to be investigated. Further, the evaluation of boneseed litter using field soil in a greenhouse may better replicate the effects of allelopathy in the field.

As a continuation of our previous research into the study of allelopathic potential of boneseed, we aimed to identify and assess the phytotoxicity of specific phenolic compounds from boneseed with a particular focus on the impacts of the concentration in the extracts, the types of extraction media and the effects of decomposition in soil over time. In addition, we aimed to evaluate the impact of boneseed litter leachate and litter-mediated soil in relation to the germination and early seedling growth of native species.

## Materials and Methods

### Sample and seed collection

We acquired permission from Parks Victoria to collect boneseed from the You Yangs Regional Park, Victoria (37^°^ 59^'^ 44^"^ S, 144^°^ 24^'^ 39^"^ E). The You Yangs site has been recognized since 1940’s as of one of Australia’s densest boneseed populations [34]. Samples for identification of phenolics including fresh boneseed organs (leaf, stem and root) and litter were collected in June 2014, while samples (boneseed plants, and boneseed non-infested soil) for other experiments were collected during July–September 2013.The samples were sealed in plastic bags and immediately transported to the laboratory. The root samples were cleaned with water to remove soil. All organs were separately chopped into 1–2 cm pieces. The samples (boneseed organs, soil and litter) were dried in air to constant weights. We used air dried samples at ambient temperatures to avoid temperature-dependant alteration of the phenolics as previously described [20, 35, 36]. Dried boneseed organs were ground in a standard commercially available coffee grinder (model no. PCML2012, Homemaker, made in China for Kmart Australia Limited), passed through a 0.5 mm mesh sieve and stored in sealed plastic vials until chemical analyses and experiments were conducted. Extraneous materials were taken out of the dried litter which was then chopped into <0.5 cm pieces and preserved in sealed plastic bags until used. Air-dried soil was passed through a 1 mm mesh sieve and stored in sealed plastic vials until all experiments were executed. Seeds of *I*. *axillaris* and *X*. *bracteatum* were collected from the wild and preserved in sealed plastic vials.

### Identifying and quantifying phenolics in boneseed

Phenolic compounds were identified using high performance liquid chromatography (HPLC) (reverse phase). Briefly, 300 mg of powdered tissue was mixed with 8 mL 50% acidified methanol (prepared with 1.2 M HCl) to homogenise on a roller mixer at 4°C for 24 h. The same procedure was repeated to prepare aqueous extracts using dH_2_O in place of acidified methanol. All the extracts were centrifuged at 4°C and 17,404 g for 12 min. The supernatant was double filtered through 0.2 μm phenomenex regenerated cellulose (RC) membrane to prepare it for HPLC analysis. Known phytotoxic phenolic compounds (gallic acid, catechin, epicatechin, p-coumaric acid, ferulic acid, phloridzin, arbutin, hydroxymethylfuraldehyde (HMF), gentic acid, chlorogenic acid and rutin) that fall within the capacity of the HPLC column [37] were included for potential identification in boneseed. After a few trials, the presence of catechin, p-coumaric acid, ferulic acid and phloridzin was confirmed and subsequent analyses concentrated on only those four compounds. Standard phenolic compounds were purchased from Sigma-Aldrich (Australia) to prepare standard stock solutions of 12.5, 25, 50 and 100 μg/ mL. A Kinetex PEP 5 μm 100 Å LC Column 250 × 4.6 mm in an HPLC system (Shimadzu, Tokyo, Japan) was used in the identification process. The mobile phase A (0.1% phosphoric acid in water) and B (0.1% phosphoric acid in acetonitrile) were fixed at a flow rate of 1 mL/min and 65 min run-time. The gradient elution profile was: 0–30 min, 90:10 (A:B); 30.01–40 min with a linear decrease of A and increase of B to 50:50 (A:B); 40.01–45 min, 10:90 (A:B), 45–55 min with a linear increase of A and decrease of B to 50:50 (A:B), and then back to initial condition. The chemical analysis was conducted in triplicate at ambient temperature with injection volumes of 10 μL. The analysis was monitored with a photodiode array detector at 220 and 271 nm. The presence of the phenolic compounds was confirmed with retention time in samples compared with standard, and was re-confirmed through spiking with specific phenolic compounds. Based on the standard curve, the quantity (μg/ mg organ) of each phenolic compound was calculated.

### Bioassay with identified phenolic compounds

Four concentrations of catechin (5, 20, 40 and 80 ppm), p-coumaric acid (2.5, 10, 20 and 40 ppm), ferulic acid (27.5, 110, 220 and 440 ppm) and phloridzin (16, 65, 130 and 260 ppm) that cover the highest and lowest concentrations identified in boneseed organs (methanol extract) were prepared by diluting the standards in distilled water. Four mixtures were prepared containing all four of these compounds at increasing concentrations e, g., mixture 1 consisted of the lowest concentrations of each phenolic described above and mixture 4 consisted of the highest concentrations of all four compounds. To control for possible extraneous effects, the pH of all solutions was measured using Pocket digital pH meter (model 99559, Dick Smith Electronics, Australia), and neutralized using 1N NaOH solution [38]. All seeds were surface sterilized with 1.5% (v/v) sodium hypochlorite for 1 min before washing in dH_2_O [39]. Thirty seeds of *I*. *axillaris* were placed in a 90 mm Petri dish lined with two 85 mm filter papers (Advantec) moistened with 5 mL of the different concentrations of allelochemicals while dH_2_O was used as a control (0%). The Petri dishes were sealed with Parafilm and incubated in a growth chamber (Thermoline incubator, Model RI250SG, Thermoline Scientific, Australia) at 30°C /20°C (day/night) temperature and 12 h photoperiod. The suitable germination temperature for *I*. *axillaris* was determined through pilot studies. Four replicates were maintained in a completely randomized design (CRD) for each treatment. The number of germinated seedlings (radicle protrudes by ≥ 1mm) in all Petri dishes were counted daily until cumulative germination levelled off (16 days). Germination indices e.g., total germination (TG), speed of germination (SpG), speed of accumulated germination (SpAG) and coefficient of the rate of germination (CRG) were calculated along with biometric parameters including hypocotyl and radicle length and weight [20]. The complete set of experiments was repeated to confirm the impacts of phenolic compounds.

### Leachate phenolics and phytotoxicity from litter-mediated soil

Two distinct treatments e.g., 1) litter with boneseed-free field soil, and 2) soil alone was used to evaluate phytotoxicity of boneseed litter-mediated soil leachate. Litter with soil treatment consisted of 100 g soil, 2 g litter (< 1 cm size) and 80 mL dH_2_O to simulate field conditions while soil alone treatment contained 100 g soil and 70 mL dH_2_O. The contents in the pots were mixed thoroughly and placed in a greenhouse. Three replicates were maintained in a completely randomized design for each treatment with a total of 6 pots. Every second day the position of the pots was changed and dH_2_O was added to the pots to compensate for evaporative loss, if needed. After 15 days, aqueous leachates were collected. Briefly, 100 mL water was added to each treatment pot and agitated for 1 hr on an orbital shaker (Orbital Mixer EOM5, Ratek Instruments Pty. Ltd., Australia) at room temperature. The leachate was centrifuged at 941 g (Econospin 120010, Sorvall Instruments, Germany) for 20 min, and the supernatant was passed through a 0.22 μm filter. Phenolic compounds in the leachate were identified using HPLC. Leachate phytotoxicity was evaluated through a germination bioassay experiment on *I*. *axillaris* in Petri dishes as described above. The pH level of the leachate was measured and neutralized as mentioned in the previous section. Electrical conductivity (EC) of the leachates was also measured using EC meter (TPS Digital conductivity meter, 2100, TPS Pty Ltd., Brendale, Queensland, Australia) to assess whether any impacts on the test species may be due to EC levels. A germination bioassay (as described in the previous section) was conducted with three replicates (3* (dH_2_O)+(soil leachate)+(litter with soil leachate)) for 16 days at which time the germination indices were calculated and biometric parameters were measured.

### Allelopathic potential of boneseed litter-mediated soil

Growing media consisted of boneseed litter (1, 2 and 4 g/pot) mixed with 200 g of boneseed-free field soil or controls containing boneseed-free field soil with no boneseed material, both in plastic pots. Although we found 2 g litter/ 100 g soil in the field conditions, the lower two doses of litter were considered as the quantity of litter may vary with season and geographical location [31, 32]. The control pots had 90 mL water added to simulate moist field conditions while extra water (equivalent to the water absorption capacity of litter) was added to pots with litter-mediated soil. Into each pot, five pre-germinated (radicle ≤ 1 mm) seedlings of *X*. *bracteatum* were transplanted. The pots with five replicates were designed in CRD in a greenhouse during September 2013. After one week the seedlings were thinned to two in each pot. Water was added manually on every second day to compensate water loss. The experimental plants were harvested after eight weeks to measure root-shoot length and weight, leaf number, and moisture, chlorophyll and free proline content of the leaves.

To measure leaf relative water content (RWC) fresh leaves were picked from same node, weighed and placed in 10 mL water in a sealed plastic tube to incubate at 4°C for 24 h. The leaves were blotted to take turgid weight. Finally, dry weight of the leaves was recorded after 24 h incubation at 60°C. Leaf RWC was calculated following the method of Saura-Mas and Lloret [40]. To measure chlorophyll, leaves were weighed and put in 7 ml of N, N-dimethylformamide in sealed plastic tubes for 24 h in dark at 4°C. Absorbance at 647 and 664 nm was measured to calculate chl a, chl b and total chlorophyll content following the method of Inskeep and Bloom [41]. Free proline was measured following the method by Bates *et al*. [42] with slight modification. Briefly, reagent was prepared in a 50 mL volumetric flask mixing 1.25 g ninhydrin with glacial acetic acid (GAA) and phosphoric acid (6M) mixture (3:2). 100 mg of fresh leaf was homogenized in 5 mL of 3% aqueous sulfosalicylic acid and centrifuged for 10 min at 25°C at 17,404 g. 2 mL supernatant was mixed with 2 mL of reagent mixture and 2 mL of GAA. The mixture was incubated in a boiling water bath for 1 h, and then cooled in an ice bath. 4 mL toluene was added and mixed thoroughly. The chromospheres containing the toluene phase were separated and absorbance was measured at 520 nm in a spectrophotometer. Results were calculated using a standard curve of proline [42].

### Data analysis

Statistical analysis was conducted using IBM SPSS 21.0. All data were presented as mean ± standard error (SE). Prior to statistical test data were transformed as necessary. The variation of quantity of phenolic compounds between water extract and methanol extract, and between initial leachate and decomposed leachate was evaluated using independent T-Test (2-tailed). The impact of identified phenolic compounds on germination of *I*. *axillaris* was evaluated using one-way ANOVA followed by *post hoc* Dunnett’s test. The difference between the two bioassay trials with standard phenolic compounds was evaluated using independent T-Test (2-tailed). The impact of litter-mediate leachate on germination of *I*. *axillaris* compared with soil alone leachate and water treatments was evaluated using one-way ANOVA followed by *post hoc* Dunnett’s test. The effect of boneseed litter on *X*. *bracteatum* was evaluated using one-way ANOVA followed by *post hoc* Dunnett’s test. Significant differences between the means were determined at a 5% level of probability (P≤ 0.05). Linear regression was adopted to express the relationship among different parameters.

## Results

### Phenolic compounds in boneseed

**Fig 1:**
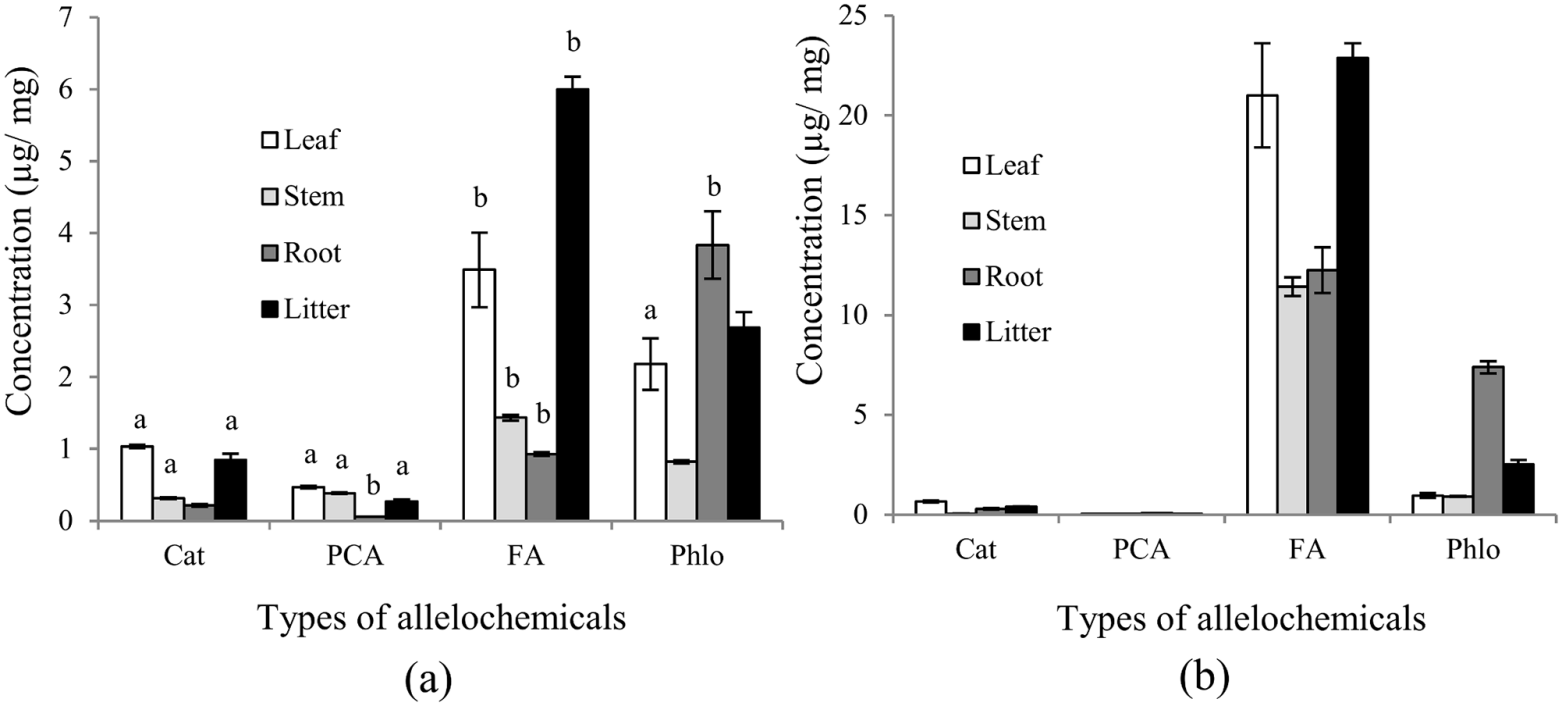
Concentrations of phenolic compounds in boneseed ((a) methanol extract and (b) water extract). The x-axis denotes the types of phenolic compounds e.g., catechin (Cat), p-coumaric acid (PCA), ferulic acid (FA) and phloridzin (Phlo). Values on y-axis denote the concentration of phenolic compounds in boneseed organs in μg/ mg. White, light ash, deep ash and black colours (filled) in the columns represent leaf, stem, root and litter extracts respectively. The error bar indicates the standard error. The letters a and b above the columns depict the significant increase and decrease of phenolics in methanol extract respectively compared with water extract while columns without letters indicate non-significant variation.

**Fig 2:**
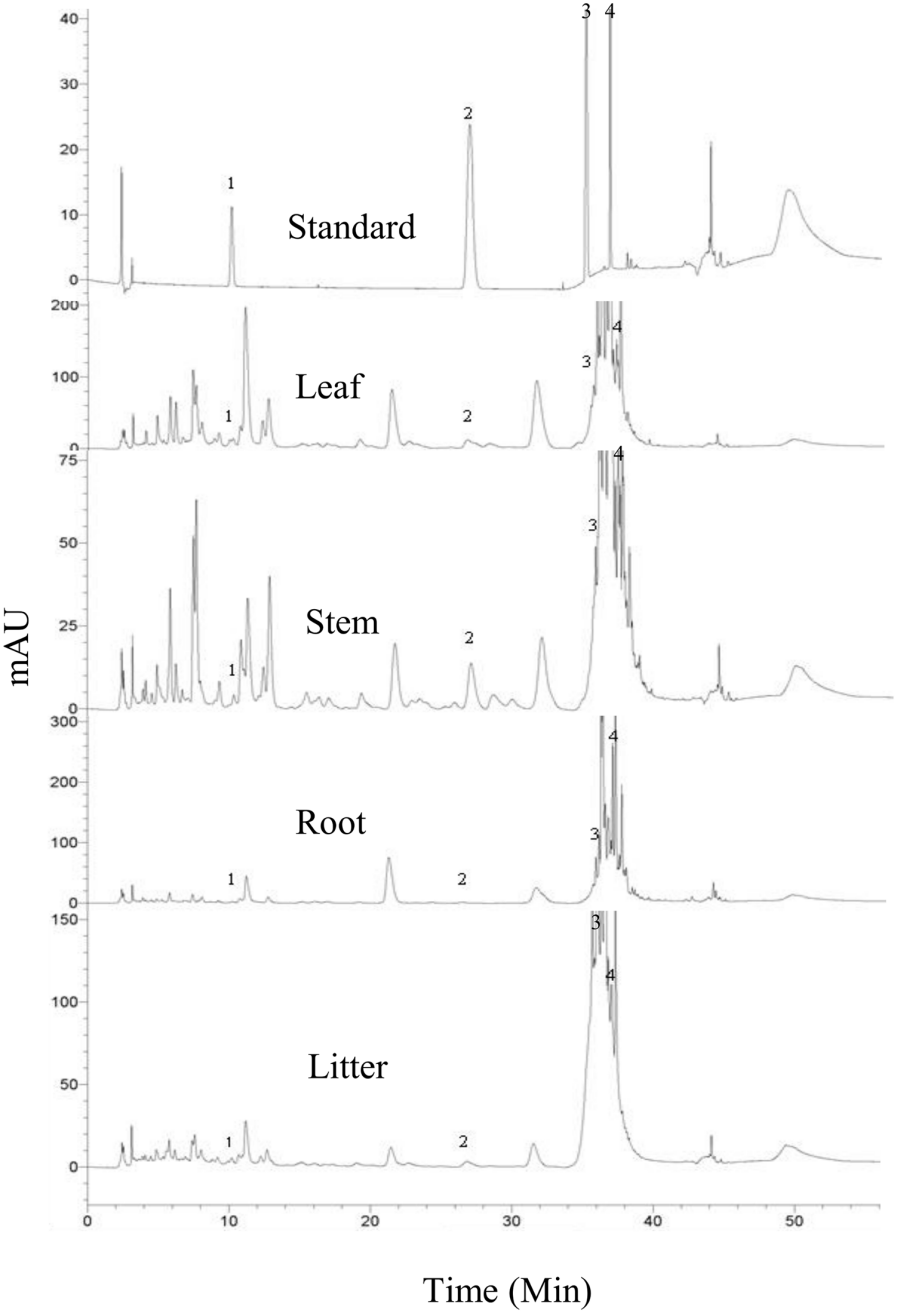
HPLC output. Chromatograms of standard phenolic compounds mixer (1 = Catechin, 2 = P-coumaric acid, 3 = ferulic acid, 4 = Phloridzin) and boneseed tissue (leaf, stem, root and litter) extracts. X-axis represents the retention time (min) and y-axis represents the unit (mAu).

Of the 13 phenolic compounds we tested, four phenolic compounds were identified in boneseed organs and litter. In methanol extracts we found the highest concentrations of catechin [1.01μg/mg (38 μg/mL)] and p-coumaric acid [0.45 μg/mg (17 μg/mL)] in leaf, the highest concentrations of phloridzin (3.36 μg/mg (126 μg/mL)] in root, and the highest concentrations of ferulic acid [5.83 μg/mg (219 μg/mL)] in litter (Fig 1). It was found that ferulic acid had the highest relative concentration (47%) followed by phloridzin (38%), catechin (10%) and p-coumaric acid (5%) (Fig 1). The HPLC output shows the original and spiked peaks of identified phenolic compounds (Fig 2, S1 Fig). Looking at the total concentrations of these four compounds revealed that litter contained the highest total quantity, followed by leaf, root and stem (Fig 1). Leaf contained 68 and 12%, 80 and 87%, 23 and 46% more catechin and p-coumaric acid than stem, root and litter, respectively (Fig 1). Litter contained 49, 76 and 84% more ferulic acid than leaf, stem and root respectively, while root contained 46, 75 and 15% more phloridzin than leaf, stem and litter respectively (Fig 1). Catechin, p-coumaric acid and phloridzin were significantly increased in methanol extracts of boneseed leaf compared with aqueous extracts while ferulic acid was significantly decreased (Fig 1). In methanol extracts of boneseed stem and litter catechin and p-coumaric acid were significantly increased compared with aqueous extracts while ferulic acid was significantly decreased and phloridzin varied non-significantly (Fig 1). The p-coumaric acid, ferulic acid and phloridzin content in methanol extracts of root were significantly decreased compared with aqueous extracts while catechin varied non-significantly (Fig 1).

### Phytotoxicity of identified phenolic compounds

The lowest doses of all identified allelochemicals had either stimulatory impacts or no impacts on germination indices (TG, SpG, SpAG and CRG) and biometric parameters (hypocotyl and radicle length and weight) of *I*. *axillaris* while other concentrations showed direct dose-response impacts to all those parameters as inhibition increased with increasing concentration (Table 1). The highest concentration of catechin, p-coumaric acid, ferulic acid, phloridzin and their mixture inhibited TG, SpG, SpAG, CRG, hypocotyl length, radicle length, hypocotyl weight and radicle weight by 7, 6, 100, 9 and 100%, 12, 7, 100, 14 and 100%, 17, 9, 100, 18 and 100%, 2, 1, 100, 2 and 100%, 15, 8, 100, 24 and 100%, 19, 5, 100, 15 and 100%, 20, 13, 100, 23, 100%, and 17, 13, 100, 22 and 100% compared with control. Although higher doses of catechin showed significant inhibition to CRG and hypocotyl and radicle length and weight compared with control, the effect on other parameters were not significant. The inhibition by p-coumaric acid was not significant for any of those parameters. Ferulic acid had the most significant inhibitory impacts on *I*. *axillaris* among the investigated phenolics with 440 μg/ mL inhibiting TG by 100%. The inhibitory effects on all parameters shown by three higher doses of ferulic acid were significant compared with control with the exception of non-significant impact on TG by second and third higher doses and SPG by third higher dose of ferulic acid. Only the highest doses of phloridzin had significant impact on CRG, and hypocotyl and radicle length and weight. All concentrations of the phenolic compound mixtures had significant inhibitory effects on all parameters of test species with the exception of the lowest dose (for all tested parameters), and the second lowest dose on TG, SpG and HL, which had no significant effect. The impact on germination indices in the repeated experiment did not significantly vary compared with first trial with the exception for the impact on SpAG (at 80 μg/ mL catechin and mixture 3) and CRG (at 40 and 80 μg/ mL catechin, 40 μg/ mL p-coumaric acid, 110 and 220 μg/ mL ferulic acid and mixture 2 and 3)(S1 Table). No significant variation of biometric parameters of *I*. *axillaris* was found in repeated experiments when compared with first trial with the exception of the significant variation in radicle length by p-coumaric acid at 2.5 μg/ mL and mixture 1 (S1 Table).

**Table 1:** Impact of standard phenolic compounds (dose response) on germination indices and biometric parameters of *I*. *axillaris*. Data presented as average ± SE. TG = Total germination, SpG = Speed of germination, SpAG = Speed of accumulated germination, CRG = Coefficient of rate of germination, HL = hypocotyl length, RL = radicle length, HW = hypocotyl weight, RW = radicle weight. The asterisks indicate the significant variation compared with control.

Treatment	Parameters							
	TG	SpG	SpAG	CRG	HL	RL	HW	RW
Control	69±4.2	2.18±0.13	12.42±0.75	7.86±0.02	3.90±0.13	8.03±0.21	0.40±0.01	0.23±0.01
Catechin (μg/ mL)								
5	70±5.3	2.25±0.17	12.95±0.95	7.92±0.04	4.19±0.07	9.28±0.29**	0.44±0.02	0.25±0.01
20	65±4.4	2.06±0.13	11.76±0.69	7.86±0.02	4.08±0.08	8.88±0.17*	0.42±0.02	0.24±0.01
40	63±4.7	1.95±0.08	10.82±0.47	7.78±0.02	3.85±0.08	7.13±0.11*	0.36±0.01	0.21±0.01
80	64±3.7	1.91±0.08	10.35±0.30	7.71±0.05*	3.30±0.07**	6.48±0.19***	0.32±0.01*	0.19±0.01*
P-coumaric acid (μg/ mL)								
2.5	70±3.6	2.34±0.13	13.76±0.91	8.03±0.04*	3.98±0.06	9.58±0.32**	0.42±0.01	0.26±0.01
10	67±1.4	2.20±0.03	12.86±0.31	7.98±0.07	4.10±0.09	9.90±0.17***	0.45±0.02	0.27±0.01
20	68±3.2	2.30±0.09	13.48±0.52	8.05±0.04*	3.88±0.10	8.53±0.31	0.38±0.01	0.22±0.01
40	65±2.2	2.02±0.08	11.32±0.48	7.80±0.03	3.60±0.11	7.60±0.20	0.35±0.02	0.20±0.01
Ferulic acid (μg/ mL)								
27.5	68±3.5	2.23±0.12	12.90±0.73	7.95±0.04	3.85±0.06	8.58±0.19	0.39±0.01	0.24±0.01
110	65±3.5	1.92±0.09	10.31±0.38*	7.67±0.03**	3.40±0.11**	7.23±0.28*	0.35±0.01*	0.19±0.01
220	63±3.6	1.60±0.08**	6.92±0.32***	7.22±0.05***	2.05±0.06***	1.50±0.09***	0.21±0.01***	0.10±0.01***
440	00***	00***	00***	00***	00***	00***	00***	00***
Phloridzin (μg/ mL)								
16.25	72±4.8	2.32±0.15	13.27±0.79	7.94±0.03	3.98±0.09	8.28±0.24	0.41±0.01	0.23±0.01
65	69±3.7	2.17±0.12	12.30±0.69	7.83±0.02	3.87±0.10	7.95±0.06	0.39±0.01	0.22±0.01
130	66±2.1	2.07±0.06	11.71±0.31	7.84±0.03	3.60±0.09	7.75±0.13	0.37±0.01	0.21±0.01
260	63±3	1.88±0.08	10.20±0.40	7.69±0.01**	2.98±0.05***	6.80±0.12***	0.31±0.01***	0.18±0.01*
Mixture								
1	68±3.5	2.25±0.06	13.23±0.16	7.95±0.06	3.98±0.09	8.03±0.19	0.41±0.02	0.23±0.01
2	63±3.6	1.88±0.10	10.15±0.50**	7.67±0.04*	3.80±0.07	6.95±0.17***	0.34±0.02*	0.18±0.01**
3	26±2.1***	0.62±0.05***	2.40±0.25***	7.07±0.04***	1.67±0.08***	1.02±0.01***	0.17±0.01***	0.09±0.01***
4	00***	00***	00***	00***	00***	00***	00***	00***

***strongly significant (*p* < 0.001),

**significant (*p* = 0.001 to < 0.01),

*poorly significant (*p* = 0.01 to ≤ 0.05),

blank means not significant.

### Leachate phenolics and phytotoxicity

**Fig 3:**
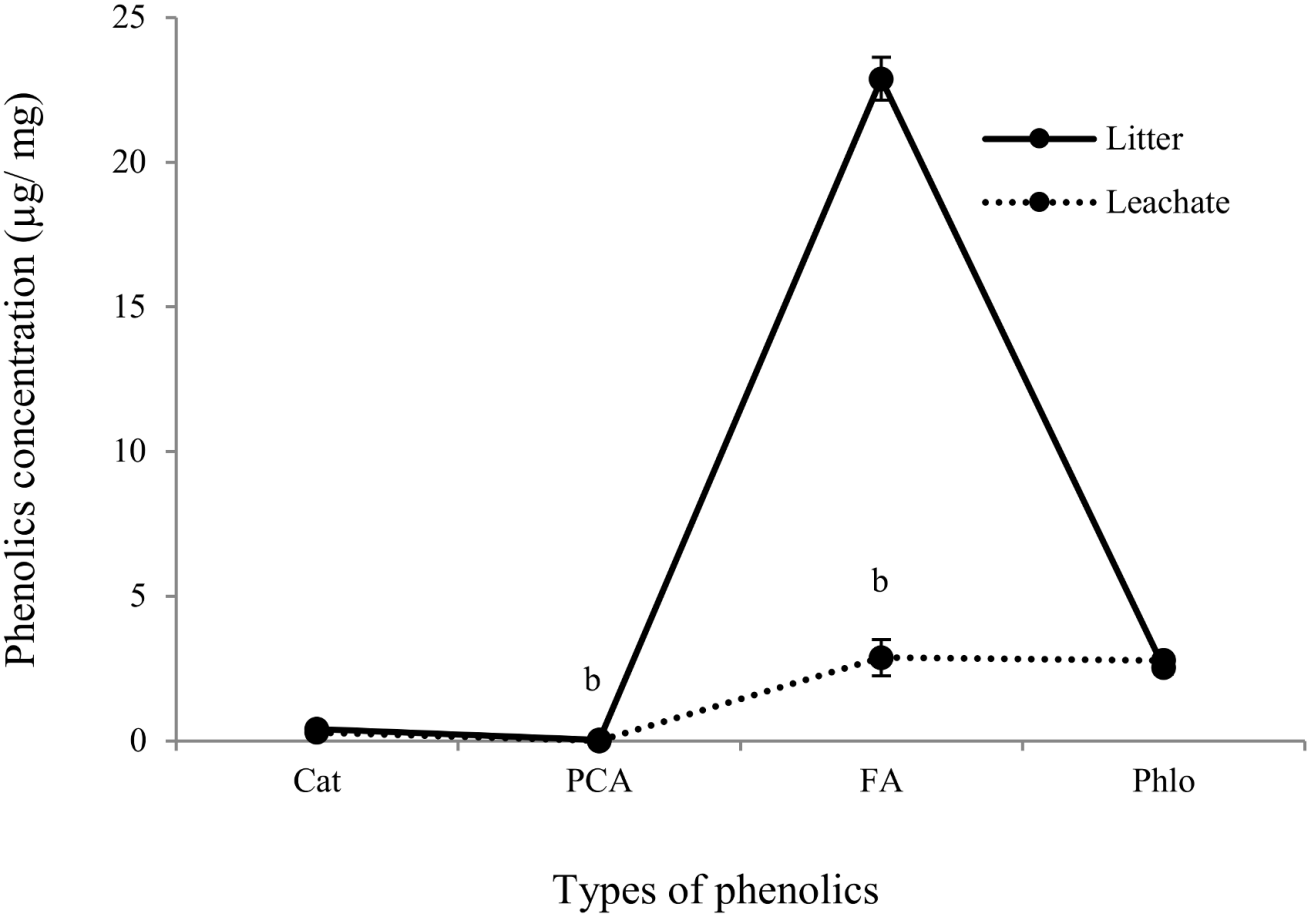
Concentrations of phenolic compounds in decomposed boneseed litter-mediated soil leachate. The x-axis denotes the types of phenolic compounds e.g., catechin (Cat), p-coumaric acid (PCA), ferulic acid (FA) and phloridzin (Phlo). Values on y-axis denote the concentration of phenolic compounds in boneseed organs in μg/ mg. The solid line denotes the phenolics in non-decomposed litter leachate while dotted line represents in decomposed leachate. The error bar indicates the standard error. The letter b on top of markers depicts the significant decrease of phenolic compounds in decomposed leachate compared with non-decomposed leachate while blank means non-significant variation.

**Fig 4:**
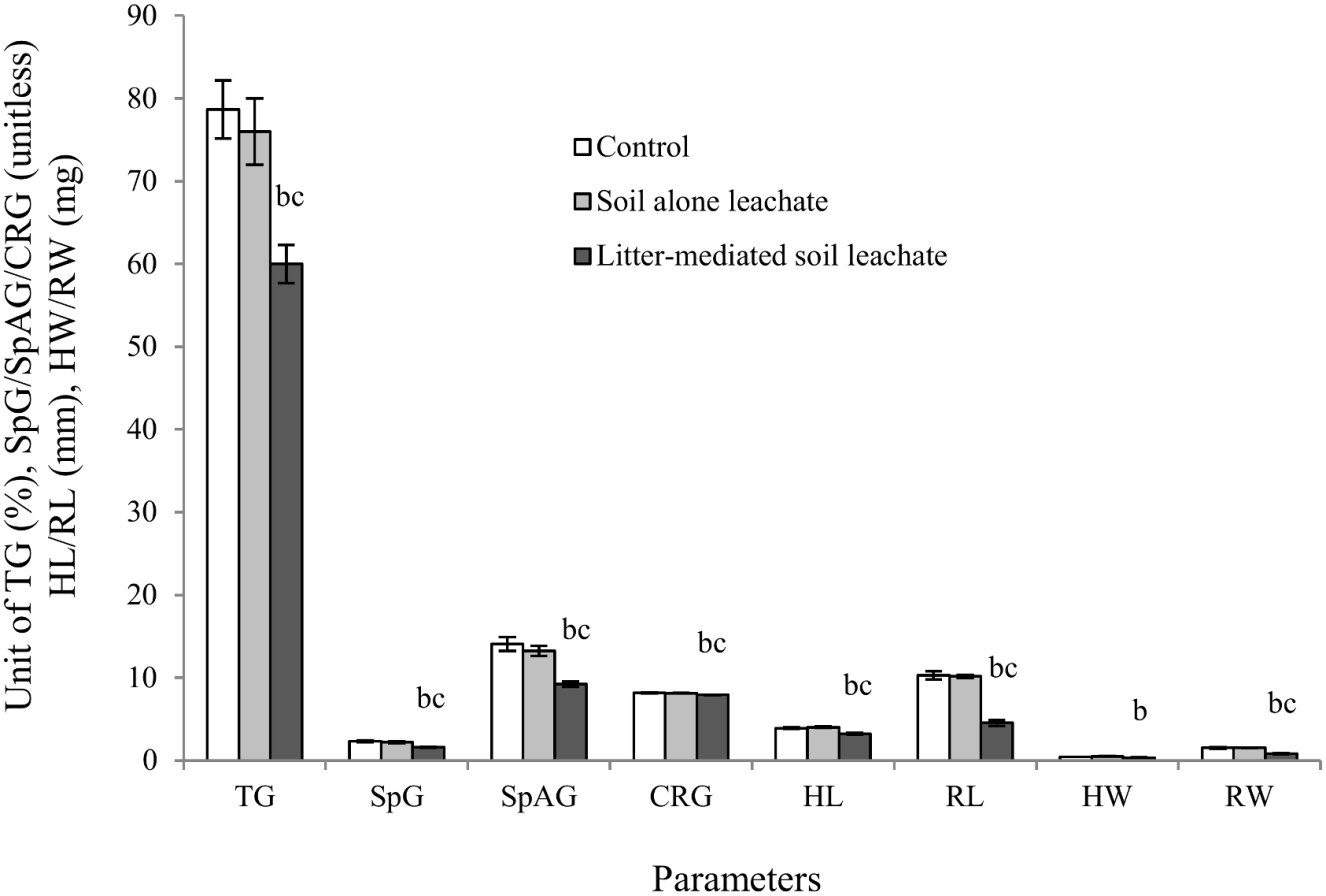
Impact of litter-mediated soil leachate on *I*. *axillaris*. The x-axis denotes the types of parameters e.g., total germination (TG), speed of germination (SpG), speed of accumulated germination (SpAG), soefficient of rate of germination (CRG), hypocotyl length (HL), radicle length (RL), hypocotyl weight (HW) and radicle weight (RW). Values on y-axis denote the units of these parameters. Data presented as average while the error bars indicate the standard errors. No filled columns, light ash colour columns and deep ash colour columns represent the impact exhibited by control, soil leachate only and litter-mediated soil leachate respectively. The letter b and c depicts significant decrease of those parameters in *I*. *axillaris* exposed to decomposed litter-mediated soil leachate compared with soil alone leachate and control respectively.

After 15 days of decomposition, p-coumaric acid and ferulic acid contents of litter-mediated soil leachate were decreased (by 100% and 87% respectively) significantly compared with their level in original litter extract (Fig 3) while the level of catechin and phloridzin did not vary significantly. Despite the reduction of identified allelochemicals in leachate after decomposition, the leachate still significantly inhibited TG, SpG, SpAG, CRG, HL, RL, HW and RW compared with both soil alone leachate (by 21%, 27%, 30%, 3%, 20%, 55, 31% and 47%) and control (dH_2_O) (by 24%, 31%, 35%, 3%, 18%, 56, 22% and 47%) (Fig 4 and S2 Table).

### Impact of boneseed litter on growth of *X*. *bracteatum*

**Fig 5:**
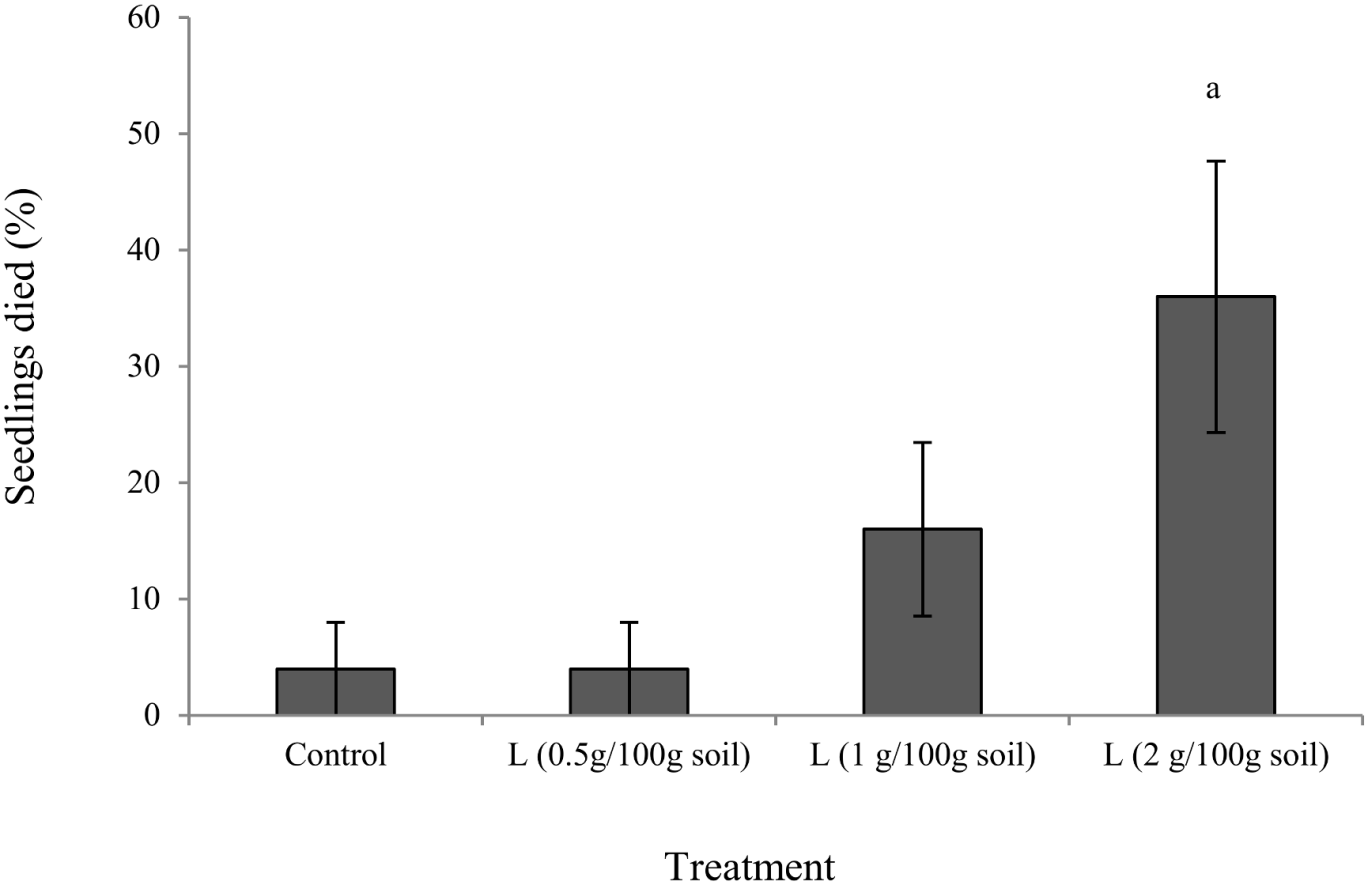
Number of *X*. *bracteatum* seedlings died due to phytotoxicity by boneseed litter-mediated soil. The x-axis denotes the treatment types (control and doses of boneseed litter (L)). Values on y-axis denote the number of *X*. *bracteatum* seedlings died after one week. The error bar indicates the standard error. The letter a indicates significant increase compared with control.

**Fig 6:**
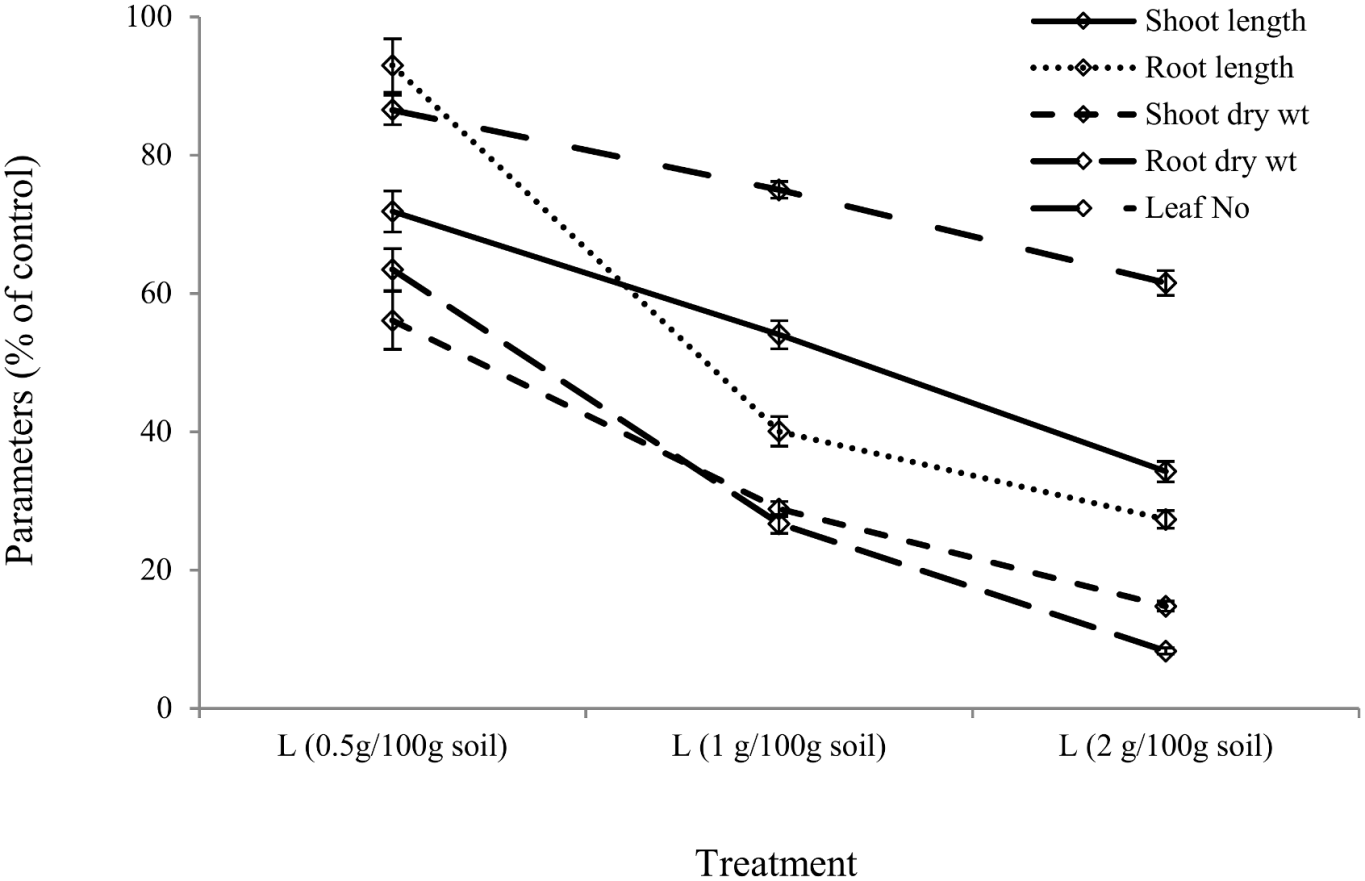
Effect of boneseed litter on biometric parameters of *X*. *bracteatum*. The x-axis denotes the doses of litter (L) in g/100gm soil. The y-axis denotes shoot length, root length, shoot dry weight, root dry weight and number of leaf of *X*. *bracteatum* (% of control). The error bar indicates the standard error.

**Fig 7:**
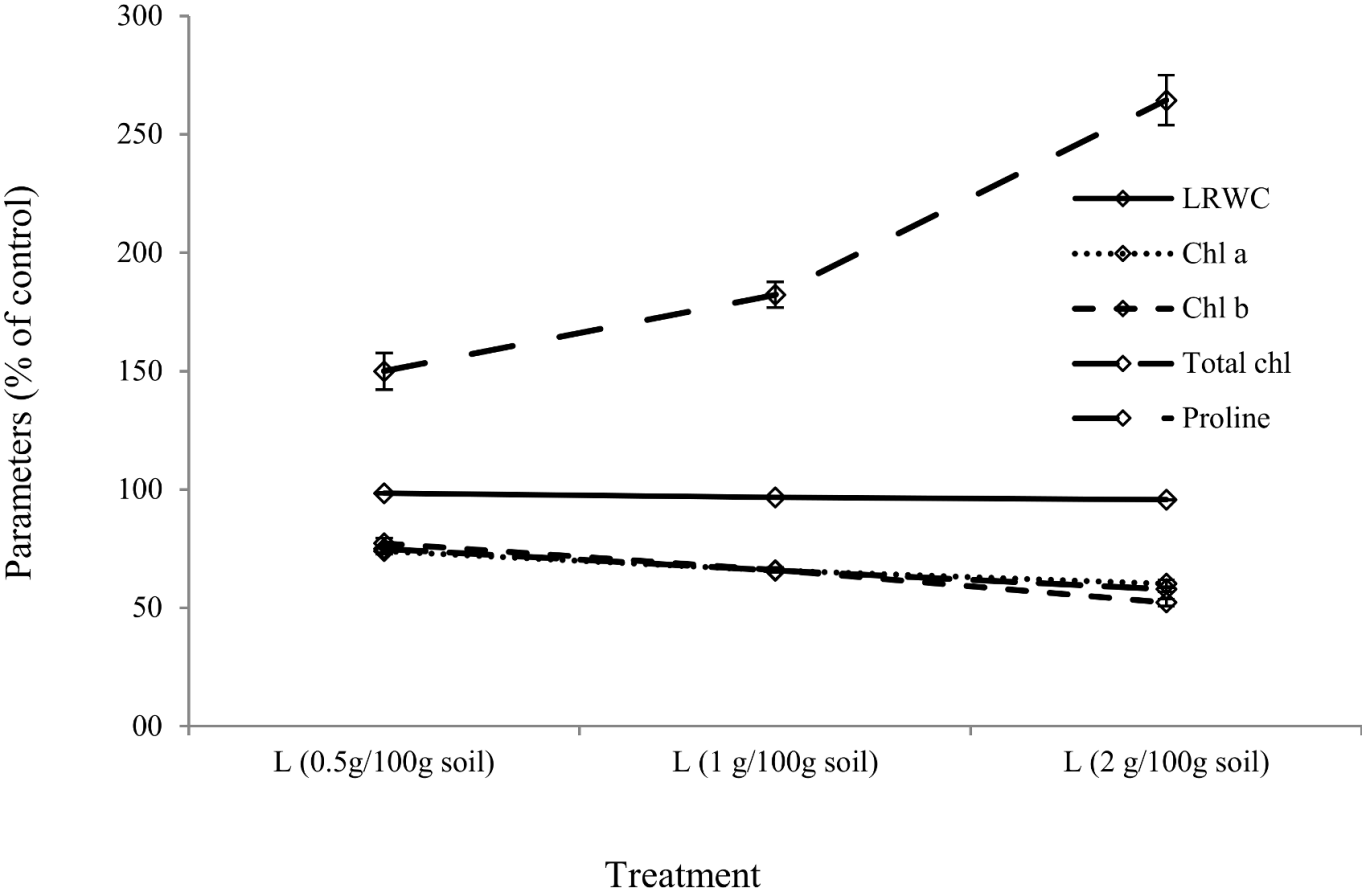
Effect of boneseed litter on biochemical parameters of *X*. *bracteatum*. The x-axis denotes the doses of litter (L) in g/100gm soil. The y-axis represents leaf relative water content (LRWC), chlorophyll a, chlorophyll b, total chlorophyll and proline content of *X*. *bracteatum* leaf. Data presented as % of control. The error bar indicates the standard error.

After one week, a significant percentage (32%) of *X*. *bracteatum* seedlings had died in the pots containing 2 g litter per 100 g soil, compared with control while the effect of litter on the number of seedlings deaths in other pots was not significant (Fig 5). 0.5 to 2 g litter in 100 gm soil inhibited shoot length, root length, shoot weight, root weight, leaf number, leaf RWC, chl a, chl b, total chl of *X*. *bracteatum* by 28–66, 7–73, 44–85, 37–92, 13–38, 2–4, 26–40, 23–48, and 25–42% respectively, when compared with the control (Figs 6 and 7) with a trend of increasing impact with increased doses of litter. Furthermore, the same comparison showed that the free proline content of *X*. *bracteatum* was increased by 50–164% compared with the control (Fig 7). One way ANOVA test exhibited that all doses of litter had significant impact on all the above parameters compared with control except for the impact on root length at 0.5 g L/ 100 g soil dose that was not significant (S3 Table). The proline content in *X*. *bracteatum* had a very strong negative correlation with its shoot length (r = −0.92) and root length (r = −0.87) (S2 Fig).

## Discussion

This study has identified four phenolic compounds (catechin, p-coumaric acid, ferulic acid and phloridzin) present in varying concentration in boneseed organs and litter. To our knowledge, these are the first identified phenolics in boneseed. The significant variation of phenolic compounds between methanol extract and water extract in this study supports our previous study that identified a significant variation of total phenolics depending on extraction media e.g., methanol, ethanol, acetone, water, etc. [20]. Although we have described the HPLC analysis of boneseed extracts in identifying phenolic compounds, more sophisticated techniques like use of polydimethylsiloxane (PDMS) in extracting specific allelochemicals in the rhizosphere may reveal novel allelopathic compounds of interest [43, 44]. Although total phenolics was not measured in the current study, we have previously shown 96.86 mg/g total phenolics in boneseed leaf (highest among the boneseed organs) [20]. The evidence of high concentration of total phenolics in boneseed when compared to other allelopathic species (about 3 times more than both *Pueraria montana* and *Phragmites australis*) [26, 45] indicates the potential of boneseed allelopathy. Ens *et al*. [19] identified volatile chemicals including phenolic compounds in bitou bush. Although the current study extended the work of Ens *et al*. [19] to identify specific phenolic compounds in boneseed, it would be of interest to examine the content of other classes of allelochemical compounds [19] in this subspecies. Similar phenolic compounds to our study have been identified by Hossain *et al*. [46] in Lamiaceae species.

The types and concentrations of allelochemicals produced by plants depend on habitat characteristics, soil type, climate, and density and phenology of allelopathic plants [47, 48]. Generally, leaves contain more allelochemicals, as we found and as has been reported in another study [49], presumably to protect against herbivores and UV radiation [50, 51]. Our study identified comparatively higher concentrations of ferulic acid and phloridzin in litter compared to leaf which is expected as degradation and transformation of phenolics commonly occurs in the presence of soil microorganisms [52].

Phytotoxicity of all four identified phenolic compounds in boneseed have been established on other test species previously [24, 25, 53, 54]. The contrasting findings of varied phytotoxicity in our study might be due to the dose response, chemical-specific and species-specific phytotoxic character of the allelochemicals [55–58]. However, further studies conducting laboratory bioassay in a more holistic way as suggested by Blum [59] may elucidate allelopathic phytotoxicity in a more ecologically realistic way. The synergistic effect of the identified allelochemicals on germination of test species was more severe than their individual impact, a finding supported by earlier studies [24, 60]. For example, mixture 3 that contained the third doses of all four phenolics had more impact on target species compared to any of the individual chemicals at the same concentrations. Mixture 3 of identified allelochemicals is equivalent to 4% aqueous extract in terms of existing concentration of allelochemicals. However, the impact of mixture 3 on the germination of *I*. *axillaris* was less than the impact shown by 2.5% aqueous extract in our previous study [20]. This suggests that the aqueous extract may contain other allelochemicals in addition to these identified four that increased the phytotoxic effect. The rank of combined concentration of four identified phenolics in boneseed organ leaf > root > stem is similar in terms of total phenolics content and allelopathic effect of their aqueous extracts [20]. However, identification of other potential allelochemicals in boneseed and their phytotoxicity assessment is important to draw firmer conclusions. The repeated bioassay with similar impact pattern on the test species ensure the phytotoxicity of identified allelochemicals. Significant variation of SpAG and CRG (for a few treatments) between first and second trials may need to be clarified by repeating the experiment. The significant variation in radicle length of *I*. *axillaris* between the first and second trials by p-coumaric acid (at 2.5 μg/ mL) and mixture 1 were not of particular interest as they were both stimulatory.

The level of allelochemicals released from decomposing plant tissues to the soil is known to decline over time, e.g., we previously identified a significant reduction in the concentration of total phenolics in leachate of boneseed litter-mediated soil (in incubator) after 40 days of decomposition [33]. This study revealed a clearer picture of decomposition of specific phenolic compounds in leachate of boneseed litter-mediated soil (in the greenhouse) after 15 days. Despite a significant reduction of identified phenolic compounds in the leachate, ferulic acid and phloridzin was remained relatively in high concentration. These higher levels of certain allelochemicals may be due to their slow rate of decomposition or microbial transformation of closely related allelochemicals as suggested in other studies [29, 61]. Microbial decomposition of phenolic compounds was also addressed by Souto *et al*. [62]. None of the identified phenolic compounds at their existing concentrations in decomposing litter-mediated soil leachate either individually or synergistically were able to inhibit germination and biometric parameters of the test species *I*. *axillaris* when compared with the bioassay experiment using standard phenolic compounds. However, further research to investigate the decomposition rate of different groups of allelochemicals over an extended period of time (at short intervals) would help to clarify their impact in the ecosystem.

This study found that leachate collected from decomposed boneseed litter-mediated soil had a significant phytotoxic effect on *I*. *axillaris*. It was confirmed that the EC level (2050 μs/ cm) we found in leachates had no adverse effects on growth of *I*. *axillaris* in agreement with Sharif *et al*. [63]. The pH should not have any impact as it was neutralized prior to bioassay experiment. Phytotoxicity exhibited by decomposing leachate applications was not due to the existence of previously identified phenolic compounds. The observed higher phytotoxicity from litter-mediated soil leachate on native *I*. *axillaris* compared with individual and synergistic effects of identified phenolic compounds in the same leachate suggests the presence of other unidentified phytotoxic compounds. The significant inhibitory effect of the decomposing litter leachate when compared to leachate from boneseed-free soil treatment (collected after same period of decomposition) and the control further supports the phytotoxic nature of decomposing boneseed litter in soil. Although the identified phenolic compounds do not seem to have a key role in the allelopathy of boneseed, however, identification and inclusion of presently unidentified allelochemicals in the litter decomposition study may shed further light on the full range of allelochemicals underpinning the phytotoxic nature of boneseed. This could be further improved through investigations on the influence that the carbohydrate, lignin, nutrients, etc. content of aqueous extracts, that generally stimulate growth [64, 65], have on their allelopathic impact. In addition, the role of microorganisms in degradation and transformation of allelochemicals in boneseed litter-mediated soil is suggested as an arena of future study. Field studies of boneseed phytotoxicity on a wider range of other associated native species are recommended.

The more naturalistic experiment (in the greenhouse) used here for identifying phytotoxicity of boneseed recognized a clear dose-response impact of boneseed litter on the early seedlings growth of *X*. *bracteatum* exemplified by clear phytotoxic effects on the radicle. The significant impact shown by boneseed litter on early seedling growth of *X*. *bracteatum* might be due to potential allelopathy of other chemical compounds rather than identified phenolic compounds as their existing concentrations in leachate had no significant inhibitory impacts. Although we have not considered the relation between allelopathy and nutrient content, Inderjit and Dakshini [66] found that nutrient released from the decomposed plant litter may play an important role in overcoming allelopathic effects. In contrast, the degradation of soil NO_3_ due to either enhancing denitrifying bacteria or suppressing nitrogen fixing or other beneficial bacteria by allelochemicals may also be responsible for the growth inhibition in target species as reported by Northup *et al*. [67] on other species. It has been found however, that interaction of soil microorganisms varies with types and concentration of specific allelochemicals [68, 69].

Litter and soil properties in boneseed and bitou bush-infested areas have been shown to inhibit germination and growth of native species by exerting various stressors [18, 70]. The very strong negative correlations between proline content and hypocotyl/ radicle length of *X*. *bracteatum* may suggest that in allelopathic-stressed condition *X*. *bracteatum* stored excessive proline as protective strategy. Similar findings have been reported in other studies [71]. The reduction of chlorophyll production in *X*. *bracteatum* in response to boneseed litter might be one of the mechanisms that retarded plant growth, similar to a finding reported by Gallardo-Williams *et al*. [9]. Although we did not directly identify the allelopathic impact on photosynthesis, the decrease in chlorophyll may reduce photosynthesis as suggested by Wang *et al*. [30]. Reduction in leaf RWC by boneseed litter may contribute in retarding growth of *X*. *bracteatum*. Further, field evidence and experimentation are imperative to demonstrate allelopathic impact in a more robust way as edaphic and environmental factors work together in influencing allelopathic effects [28].

## Conclusions

Boneseed litter contained the highest phenolic compounds (combined) followed by the leaf, root and stem respectively. The quantity of phenolic compounds extraction significantly varied with extraction media. The relative phenolic concentration in boneseed was ranked as ferulic acid > phloridzin > catechin > p-coumaric acid. The overall impact shown by the phenolics at a concentration found in boneseed organs to germination was in the order of mixture > ferulic acid > catechin > phloridzin > p-coumaric acid. It seems that identified phenolic compounds in the undecomposed litter-mediated soil leachate have a high toxic effect. However, the presence of them was significantly reduced to the nontoxic level in the decomposed leachate. Therefore, the phytotoxic effect of decomposed litter-mediated soil leachate was probably due to other chemical compounds of potential allelopathy. Further, the more naturalistic experiment with boneseed litter-mediated soil in the greenhouse exhibited significant phytotoxicity to *X*. *bracteatum* which was at least partly due to allelopathy of other unidentified chemical compounds rather than identified phenolic compounds. The reduction of leaf RWC and chlorophyll level in target species might be the partial mechanisms inhibiting plant growth induced by boneseed litter allelopathy. Storage of substantial quantity of free proline in target species may be interpreted as a response to the stressed condition due to allelochemicals. These critical findings are important for better understanding the invasive potential of boneseed and devising control strategies. Further study is needed to identify all potential allelochemicals in boneseed and decomposed leachate and to determine their phytotoxicity on associated species, particularly in field conditions. A seasonal and biogeographic study is also recommended for future study as phenolic content, microbial decomposition and phytotoxicity varied in relation to these parameters.

## Supporting Information

S1 Fig HPLC output showing chromatograms of boneseed tissue (leaf, stem, root and litter) extracts. (DOCX)

S2 Fig Correlation between free proline and shoot-root length of *X*. *bracteatum*. (DOCX)

S1 Table Impact of standard phenolic compounds (dose response) on germination indices and biometric parameters of *I*. *axillaris* (Repeated experiment). (DOCX)

S2 Table Statistical significance analysis of impact of boneseed litter-mediated soil leachate on *I*. *axillaris*. (DOCX)

S3 Table Statistical significance analysis of impact of boneseed litter-mediated soil on *X*. *bracteatum*. (DOCX)
