# Supplementary material for: Identification and Phytotoxicity Assessment of Phenolic Compounds in Chrysanthemoides monilifera subsp. monilifera (Boneseed)
Source: PLoS One. 2015 Oct 14;10(10):e0139992. doi: 10.1371/journal.pone.0139992 (PMC4605635; doi:10.1371/journal.pone.0139992)
Supplement: S1 Fig — (DOCX) [file pone.0139992.s001.docx]

S1 Fig.

Leaf extract (spiked with catechin)

Time (Min)


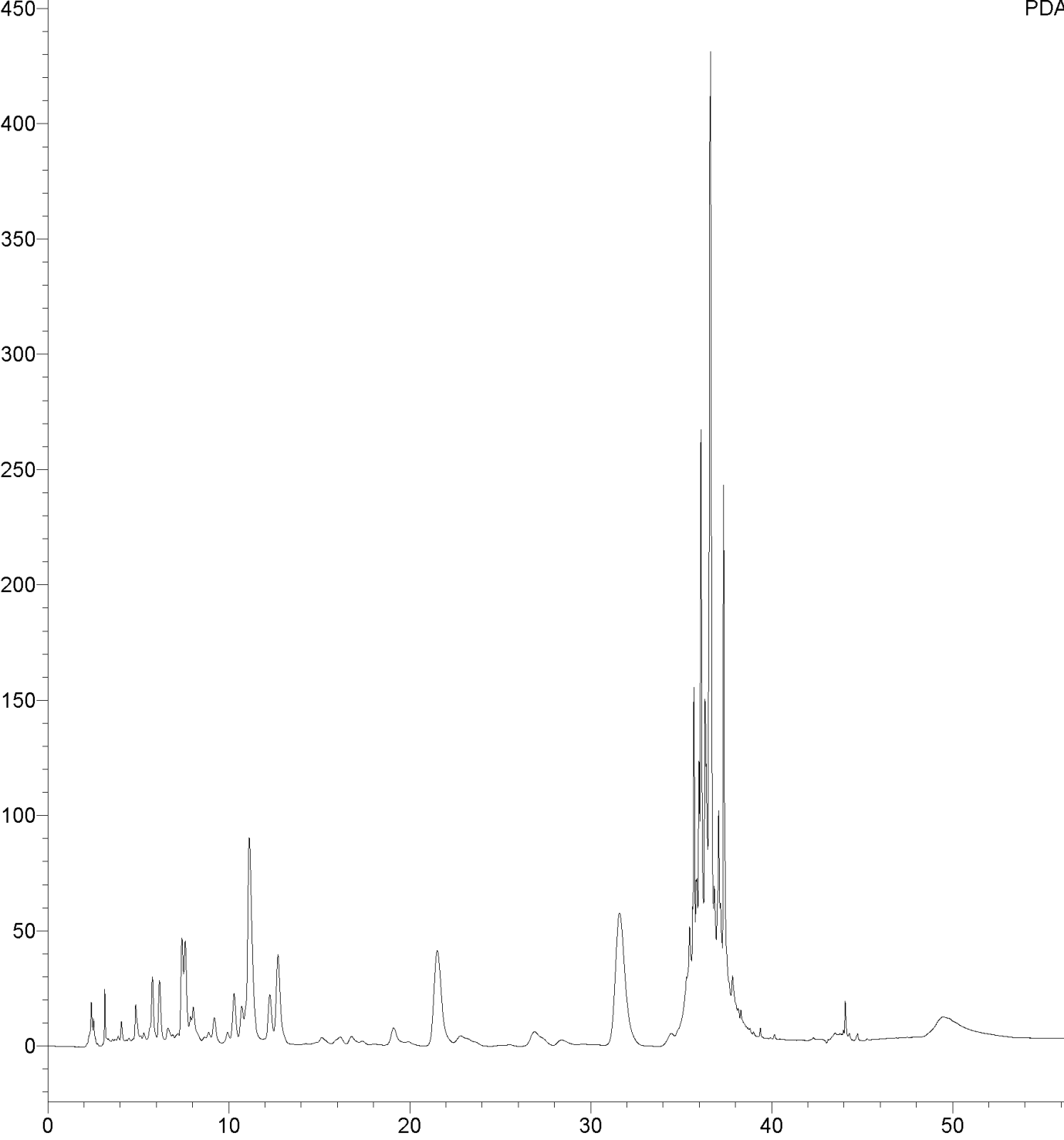


1

mAU

Stem extract (spiked with catechin)

Time (Min)


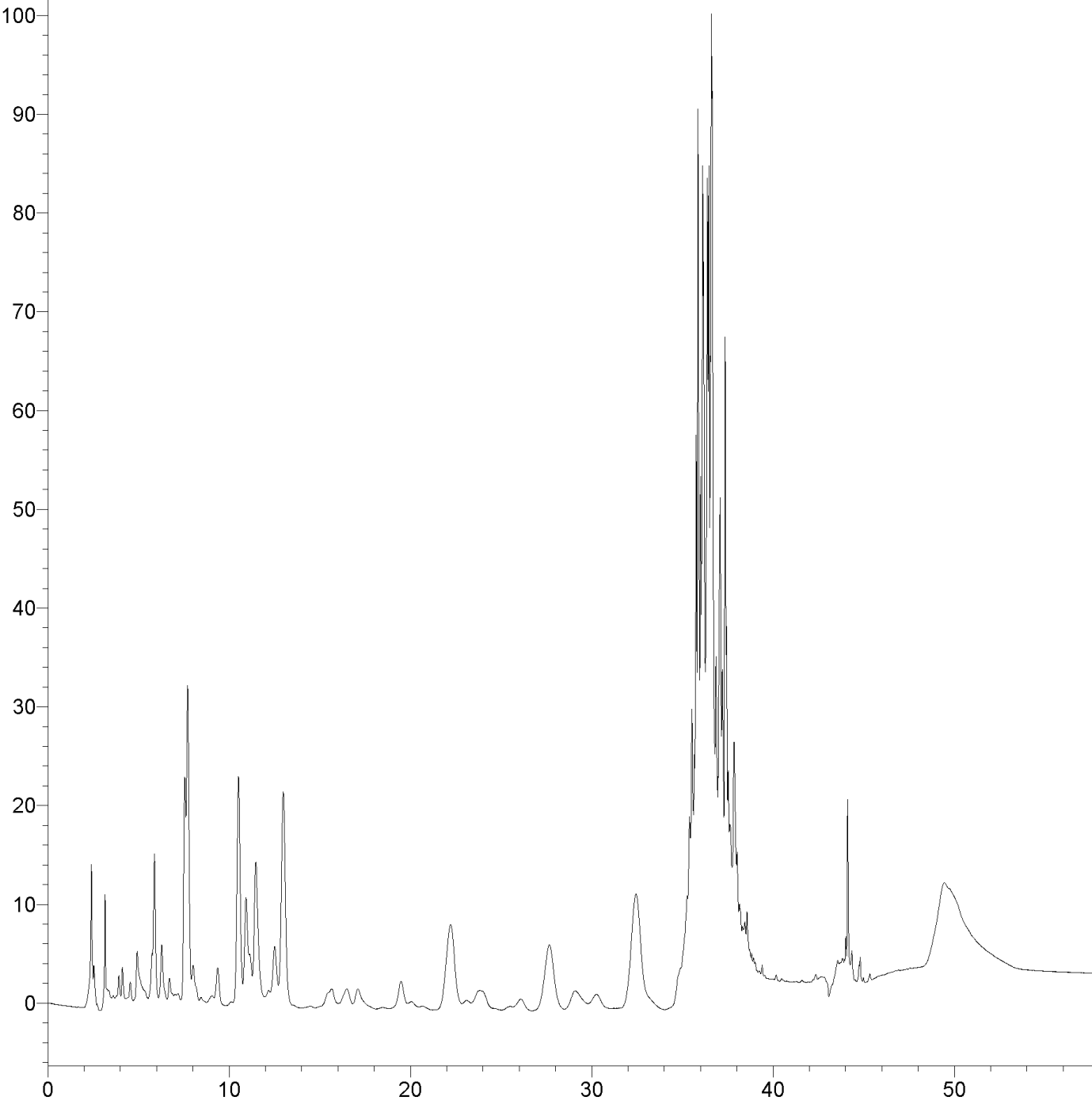


1

mAU

Root extract (spiked with catechin)

Time (Min)


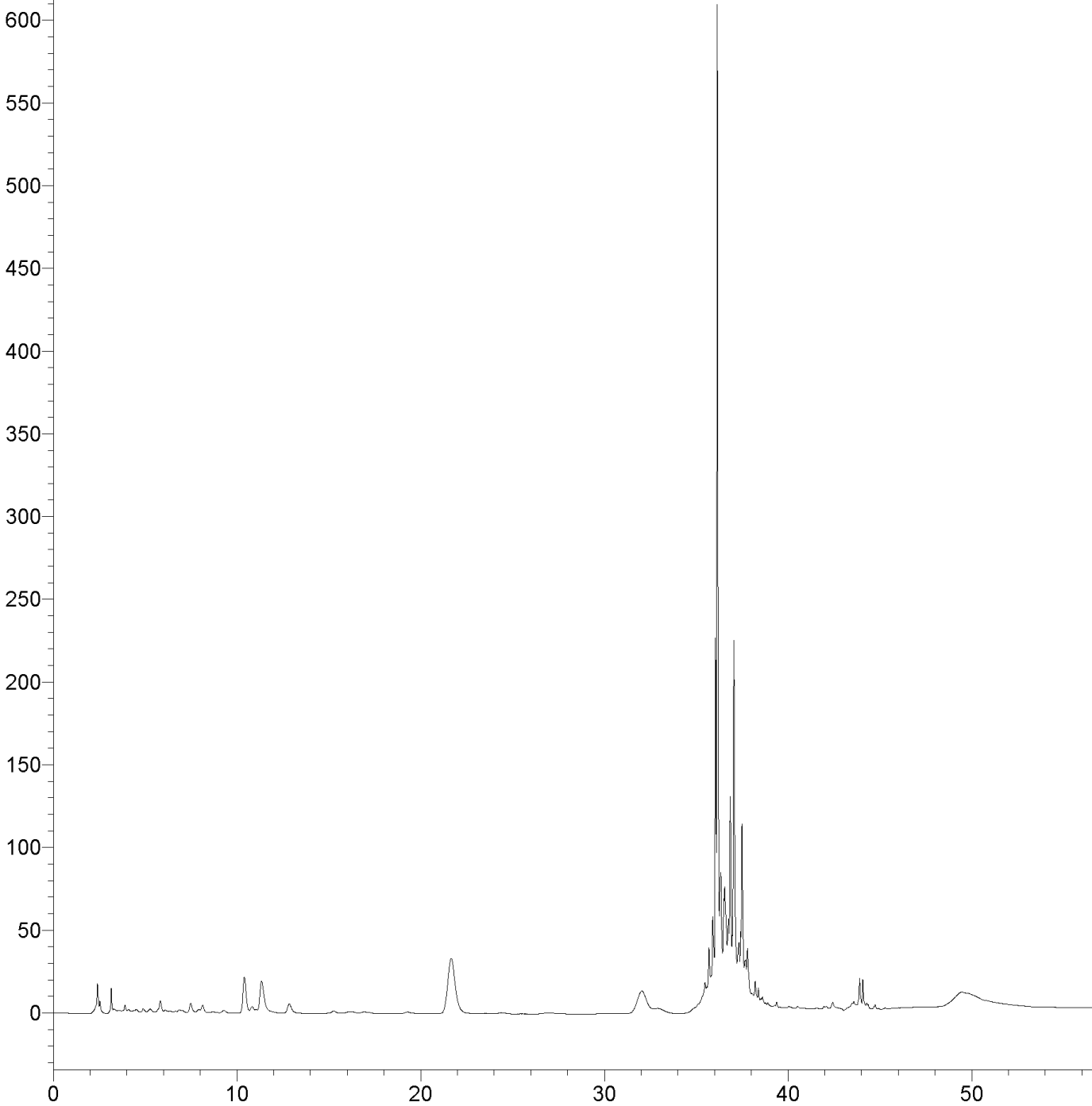


1

mAU

Litter extract (spiked with catechin)

Time (Min)


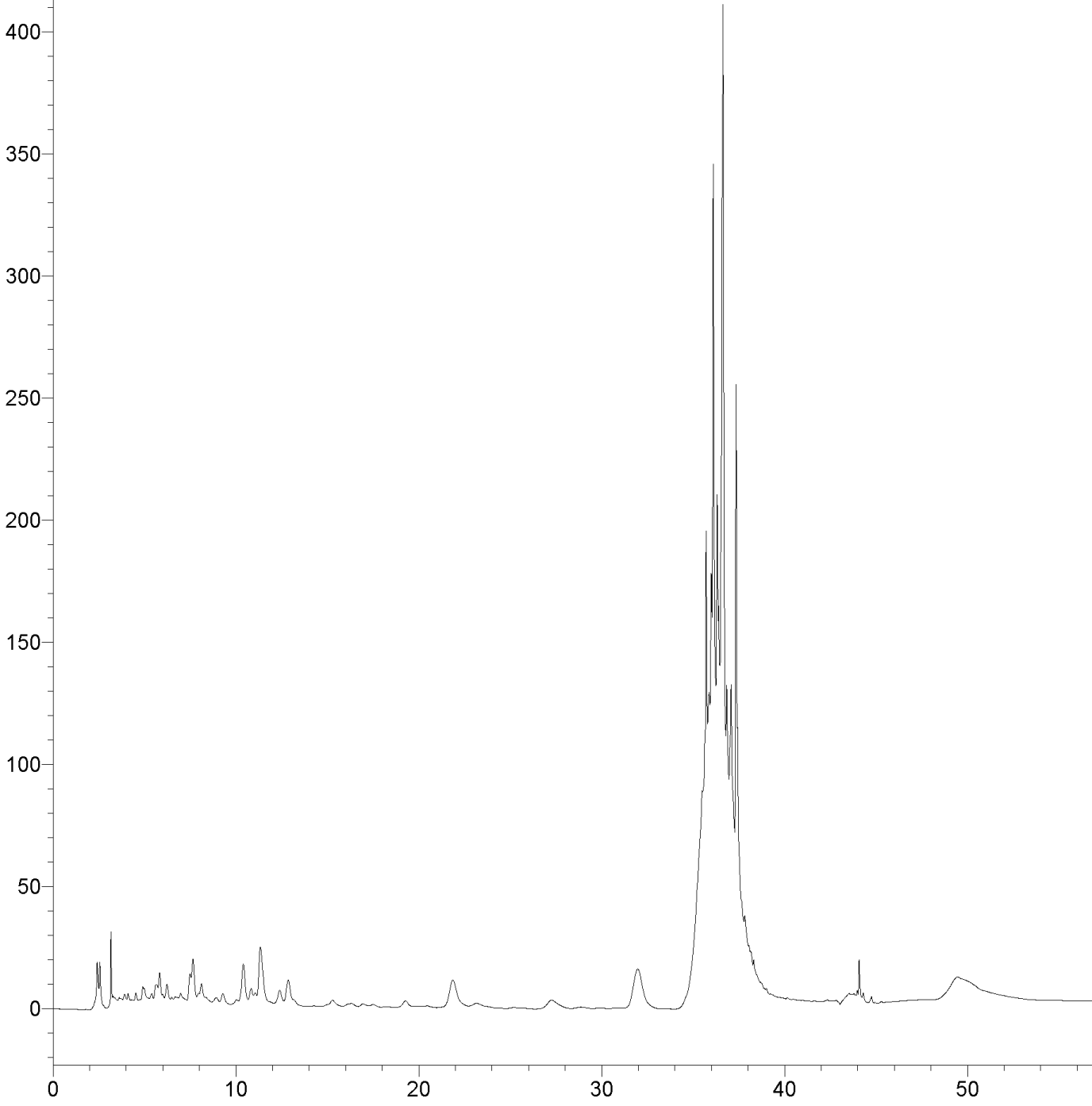


1

mAU

Leaf extract (spiked with PCA)

Time (Min)


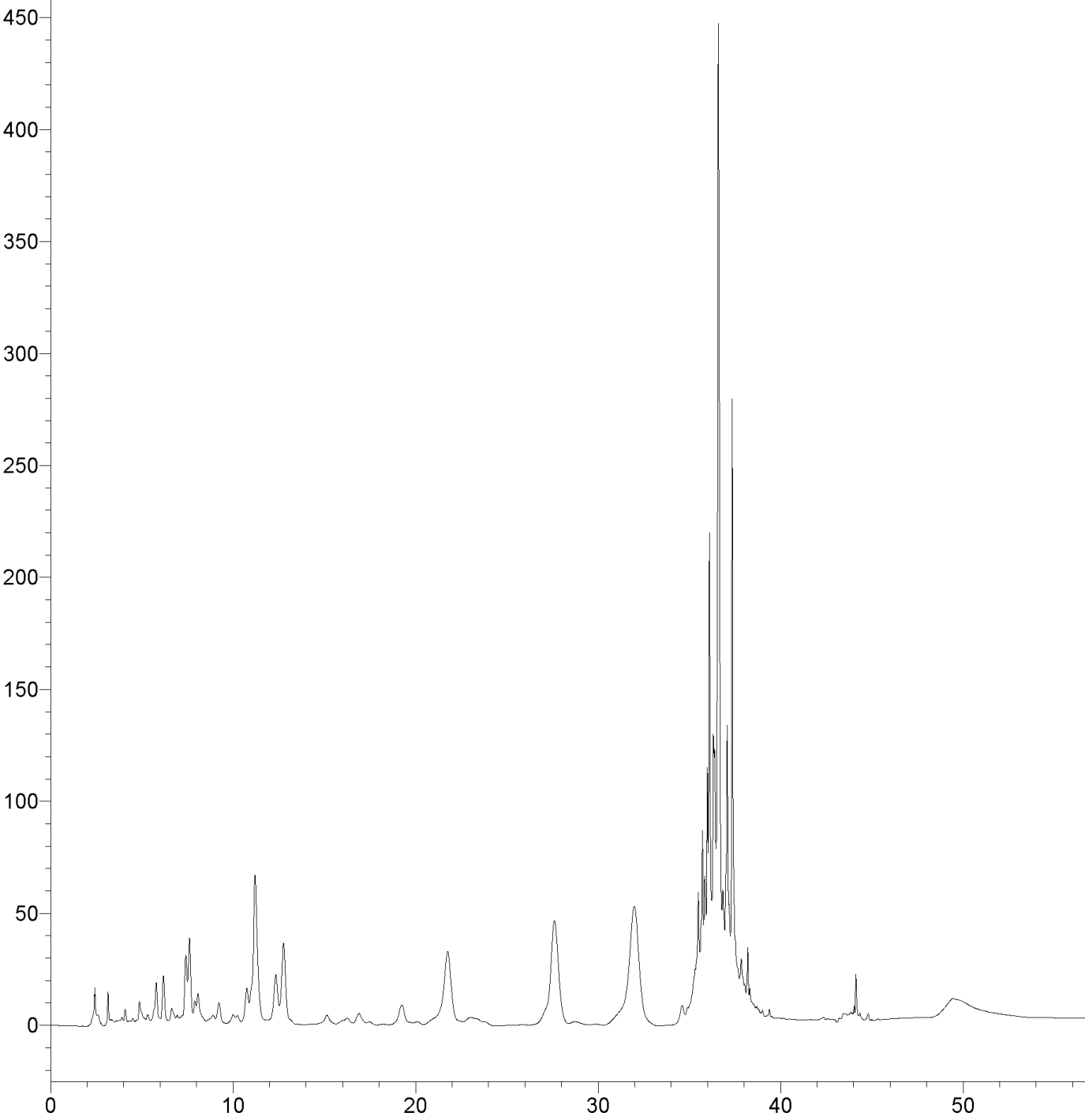


2

mAU

Stem extract (spiked with PCA)

mAU


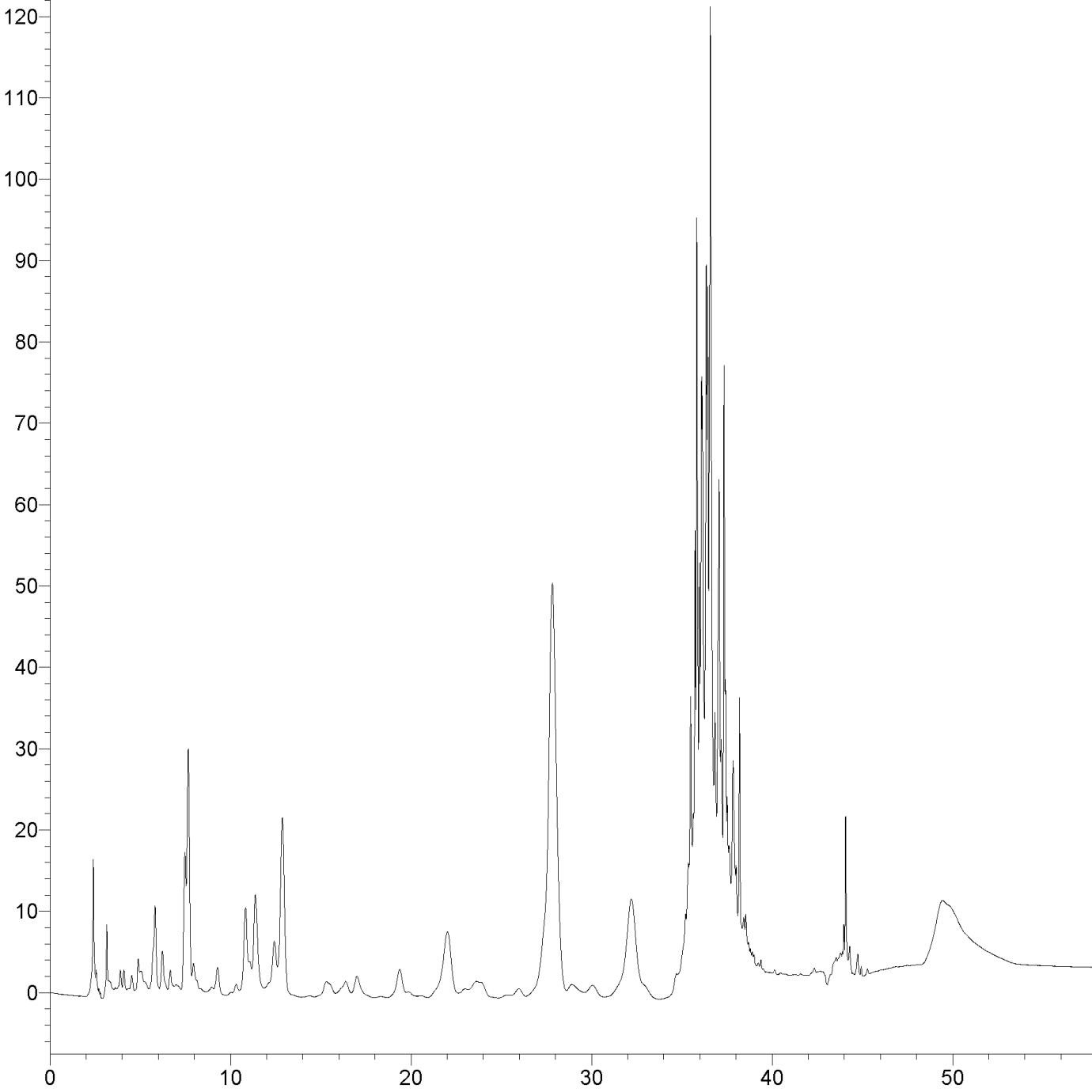


2

Time (Min)

Root extract (spiked with PCA)

mAU

Time (Min)


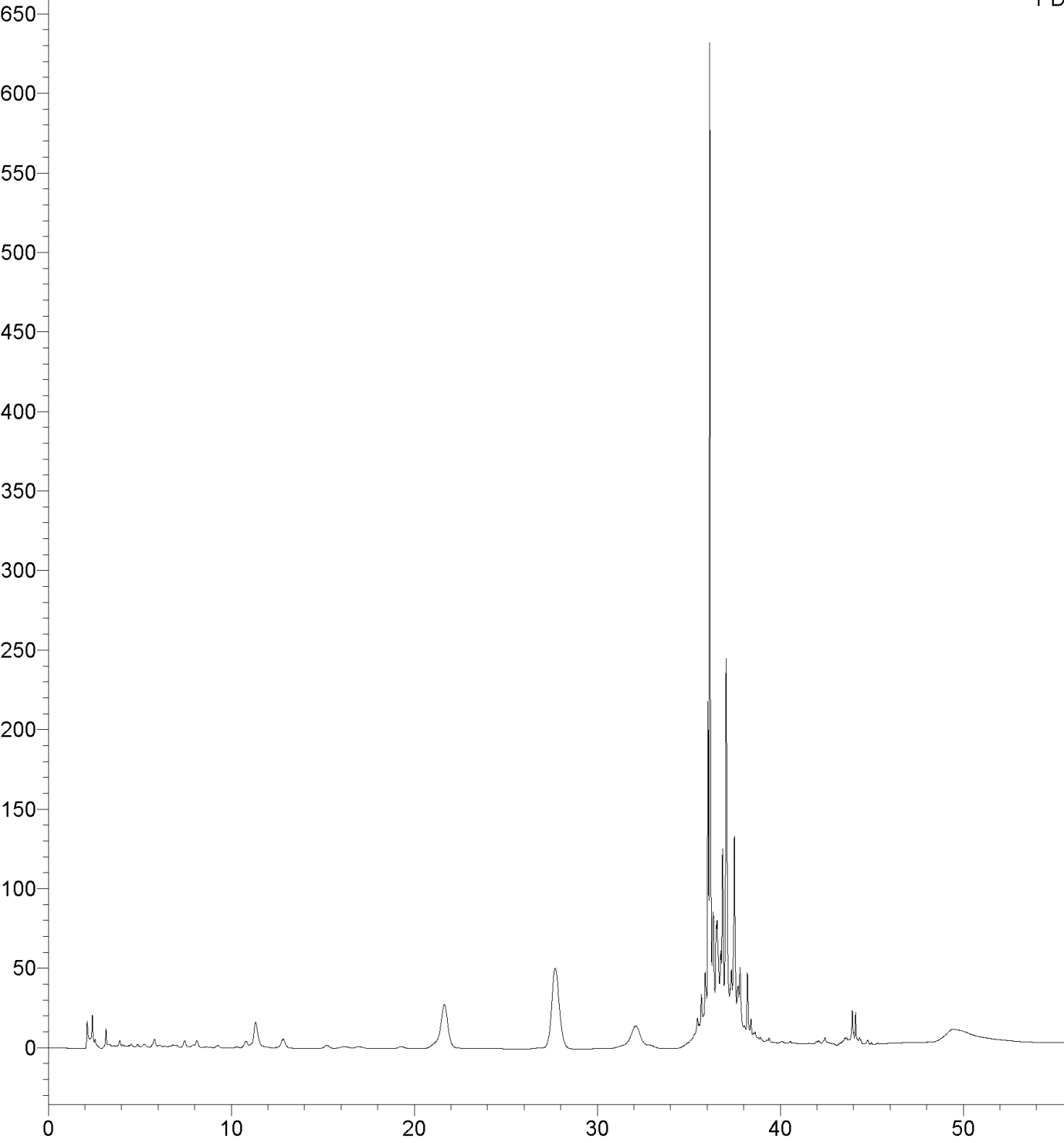


2


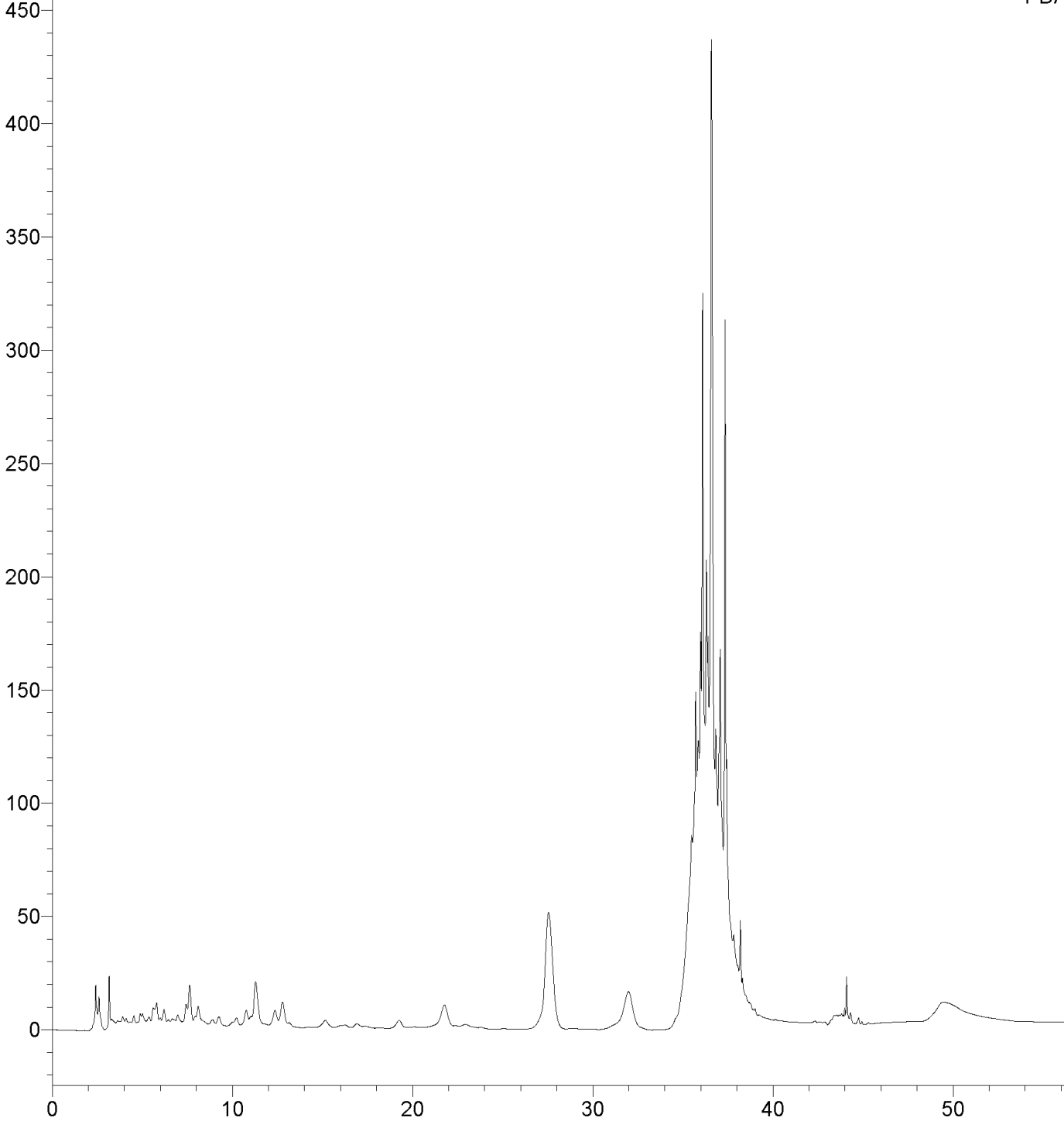


2

Time (Min)

mAU

Litter extract (spiked with PCA)

Leaf extract (spiked with FA)

mAU

Time (Min)


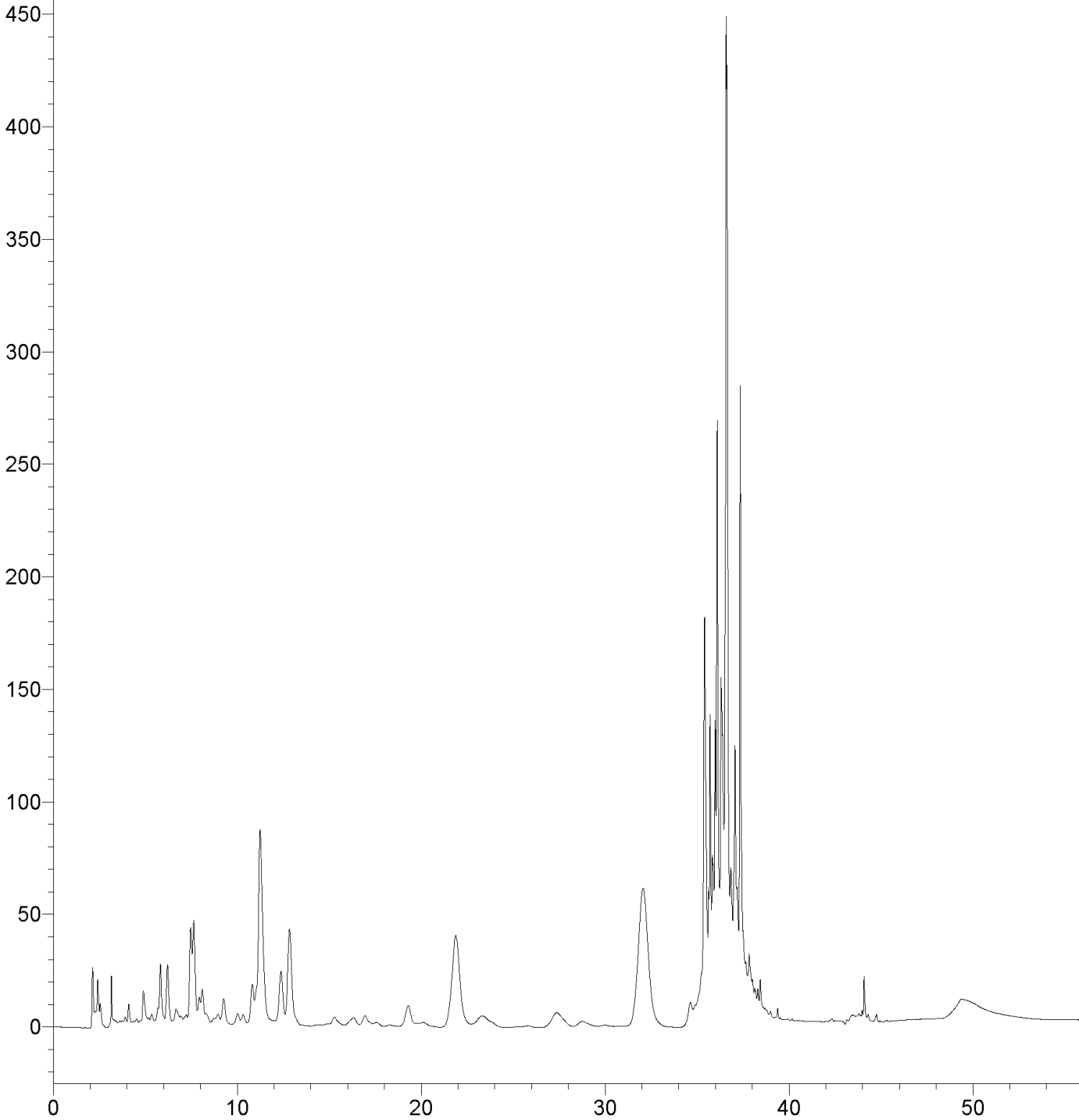


3

Stem extract (spiked with FA)

Time (Min)


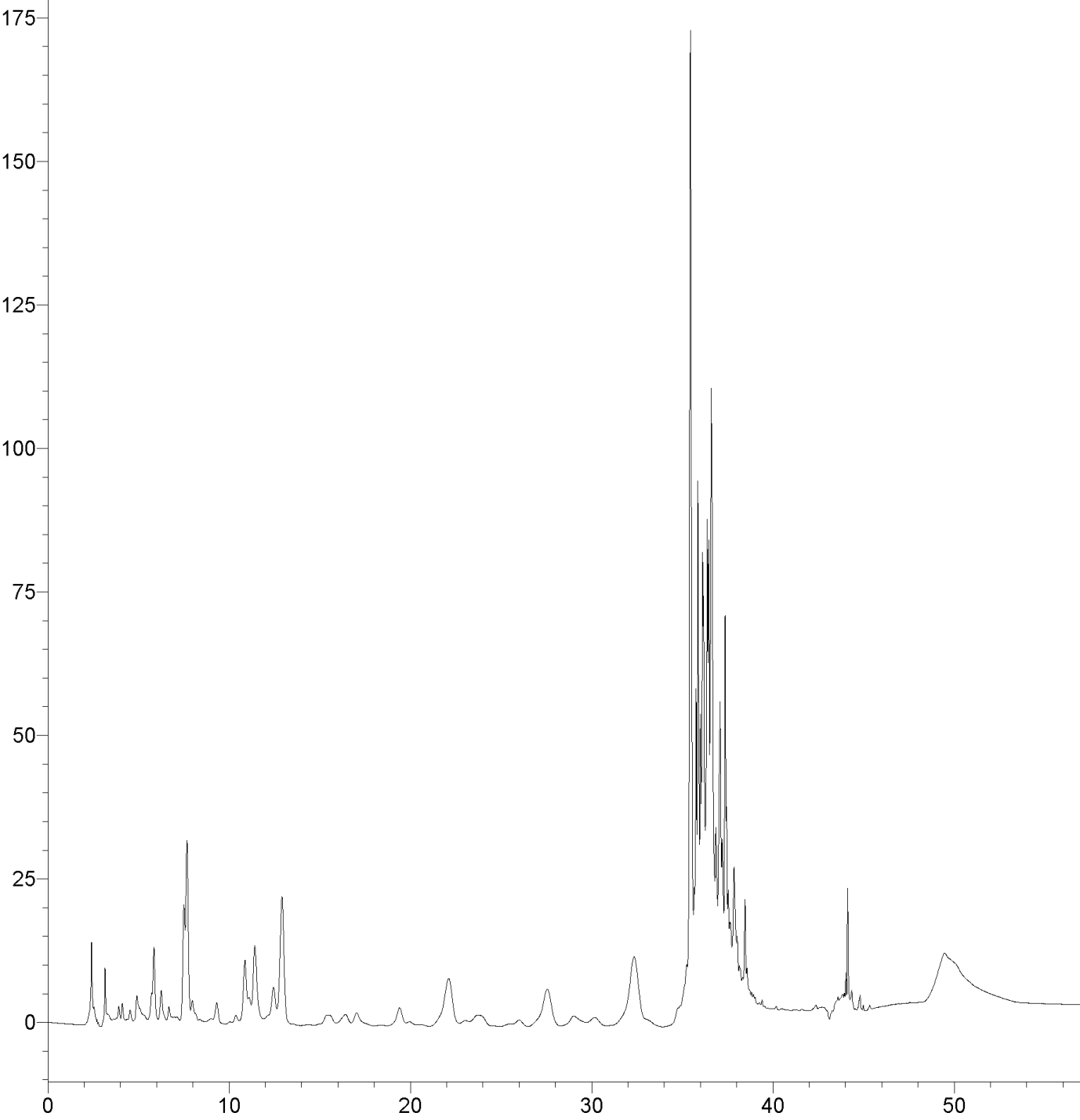


3

mAU

Root extract (spiked with FA)

mAU


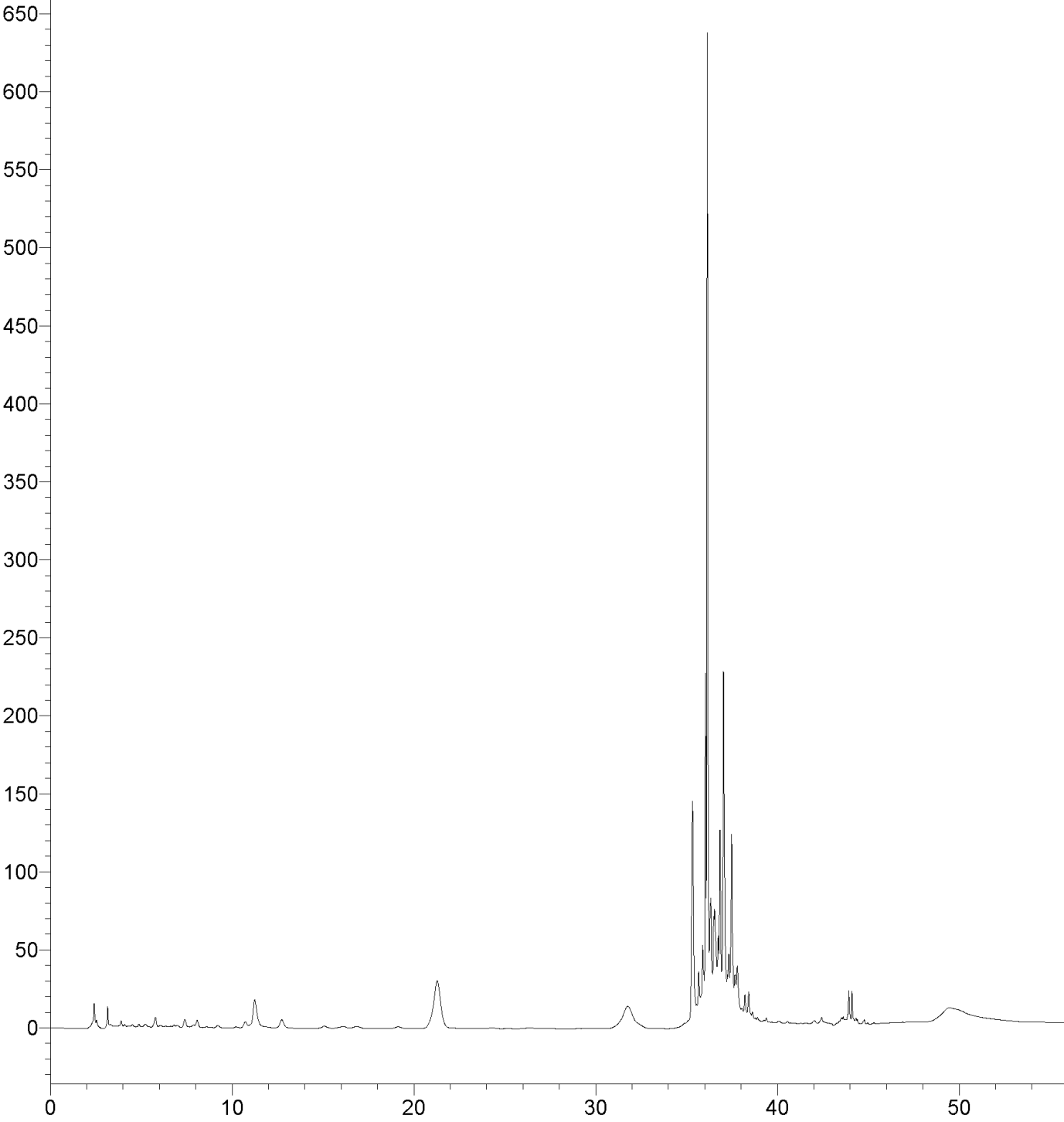


3

Time (Min)

Litter extract (spiked with FA)

Time (Min)

mAU


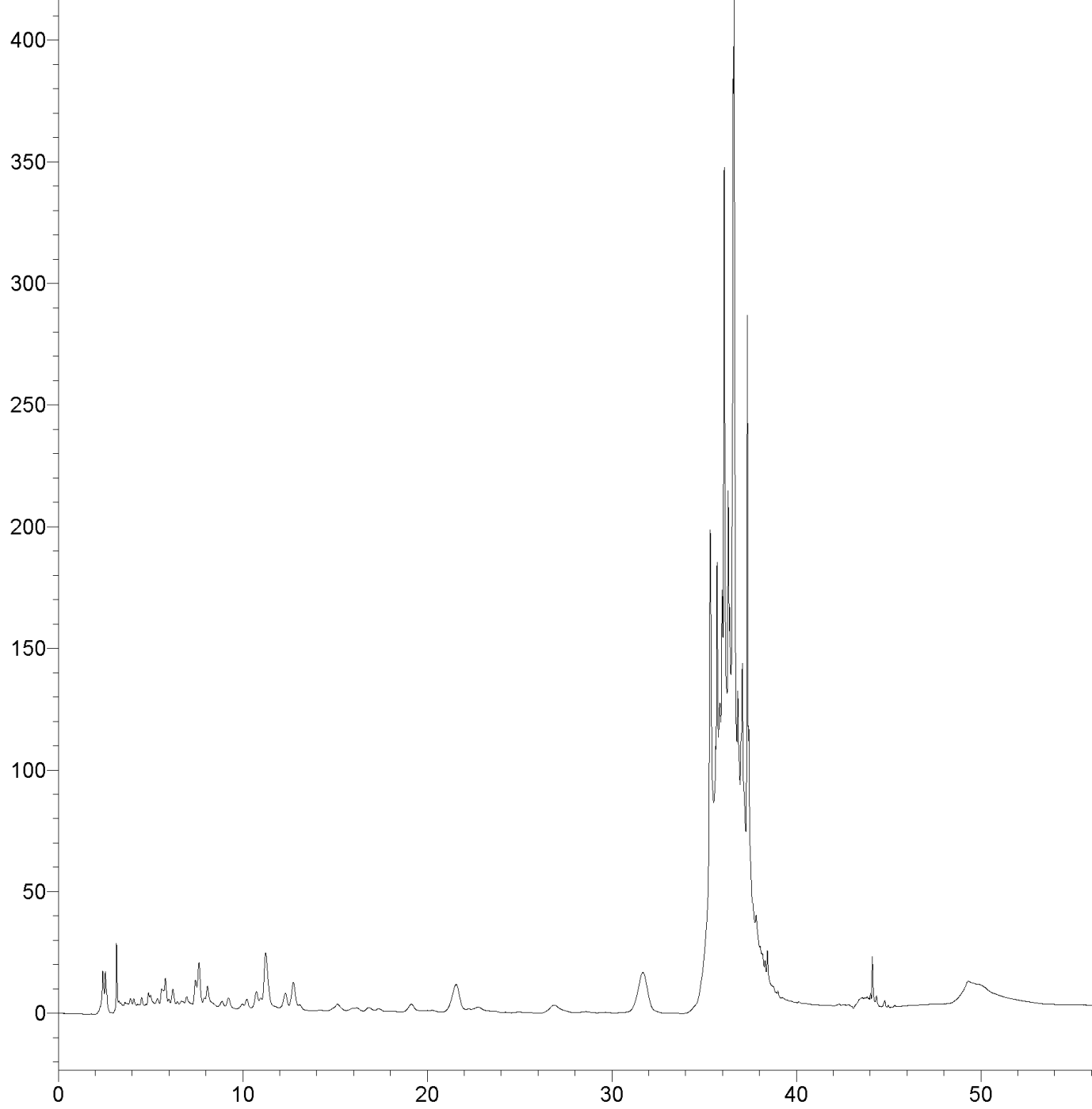


3

Leaf extract (spiked with Phloridzin)

Time (Min)

mAU


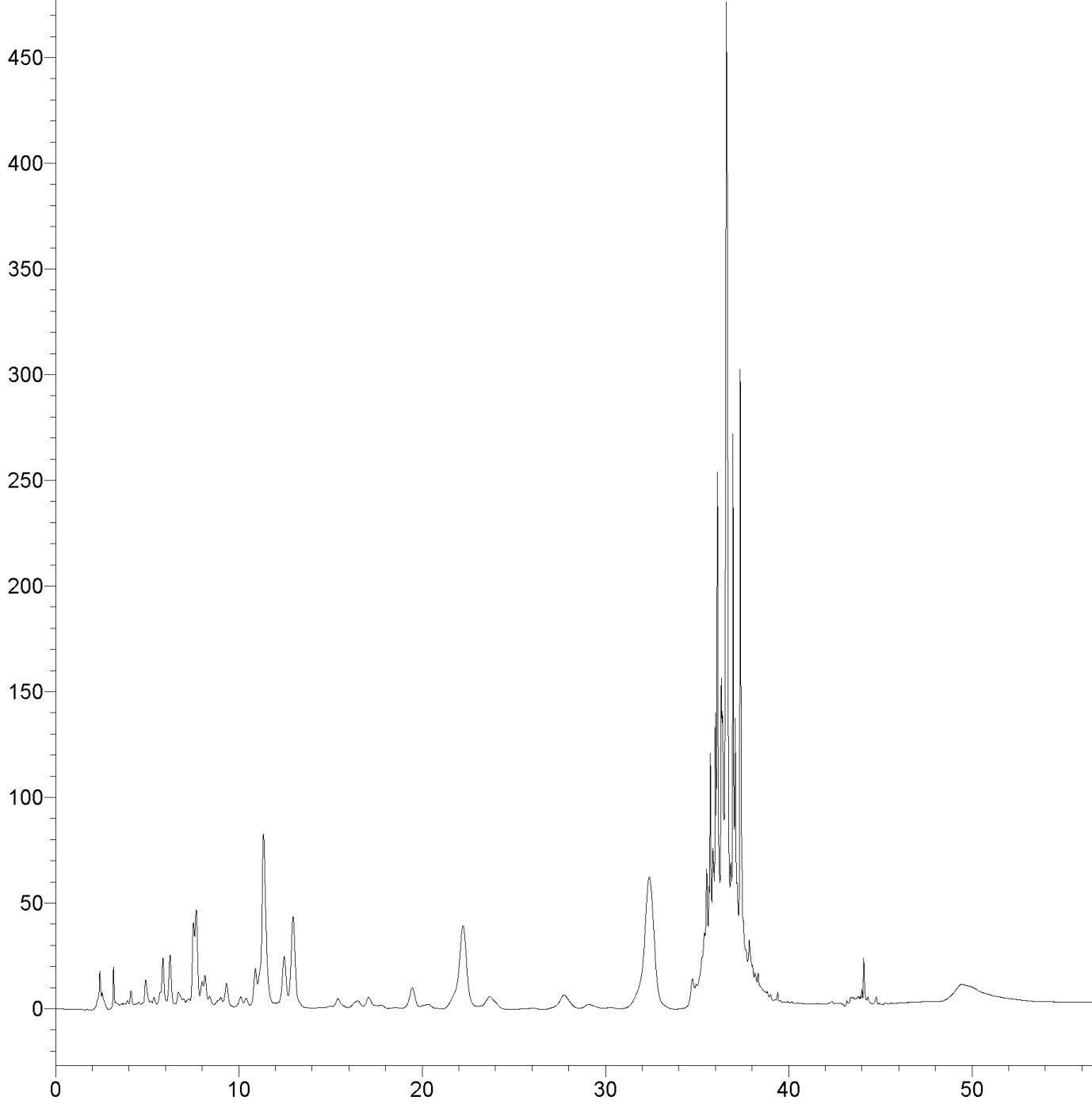


4

Stem extract (spiked with Phloridzin)


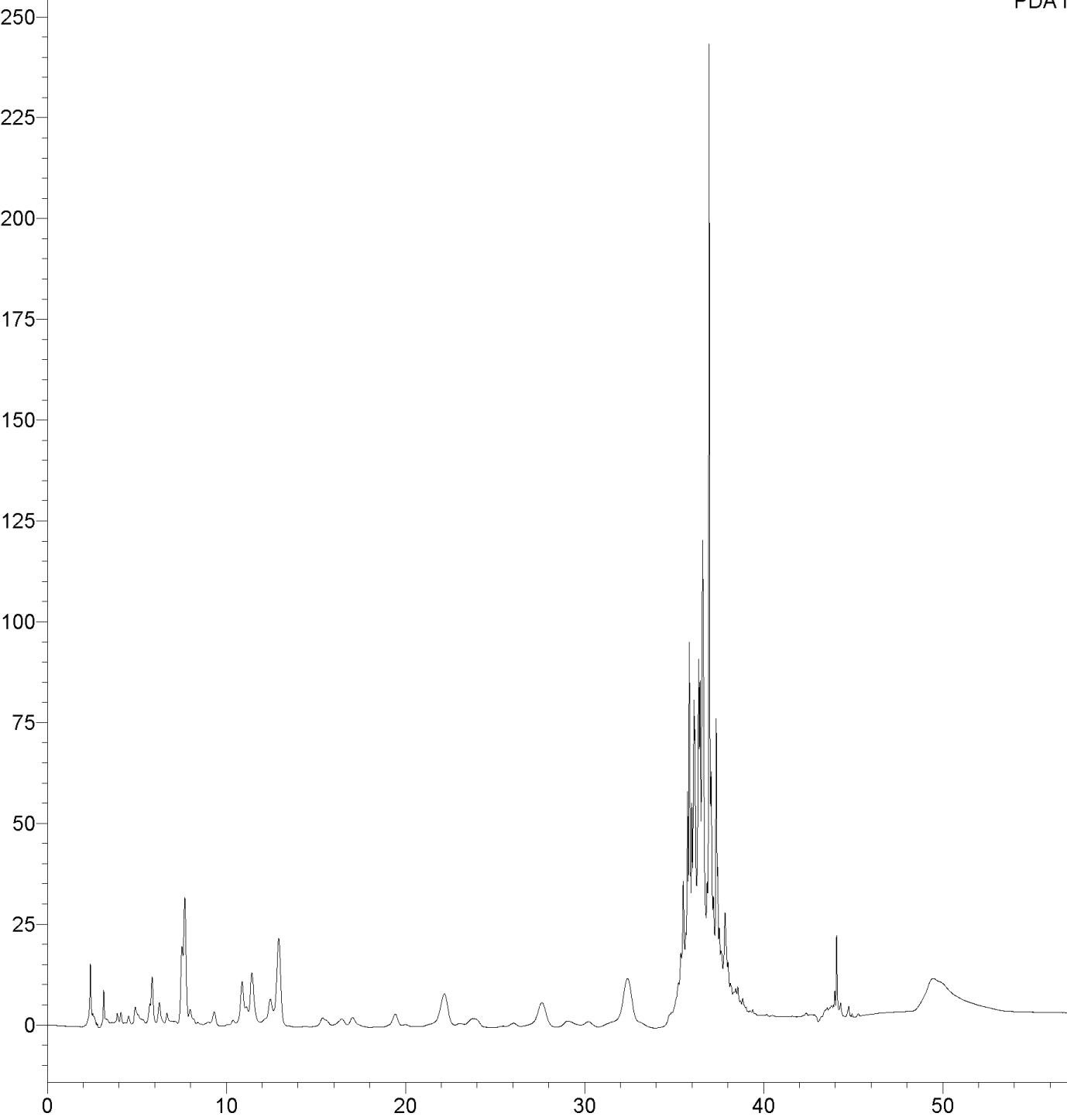


4

mAU

Time (Min)

Root extract (spiked with Phloridzin)

mAU


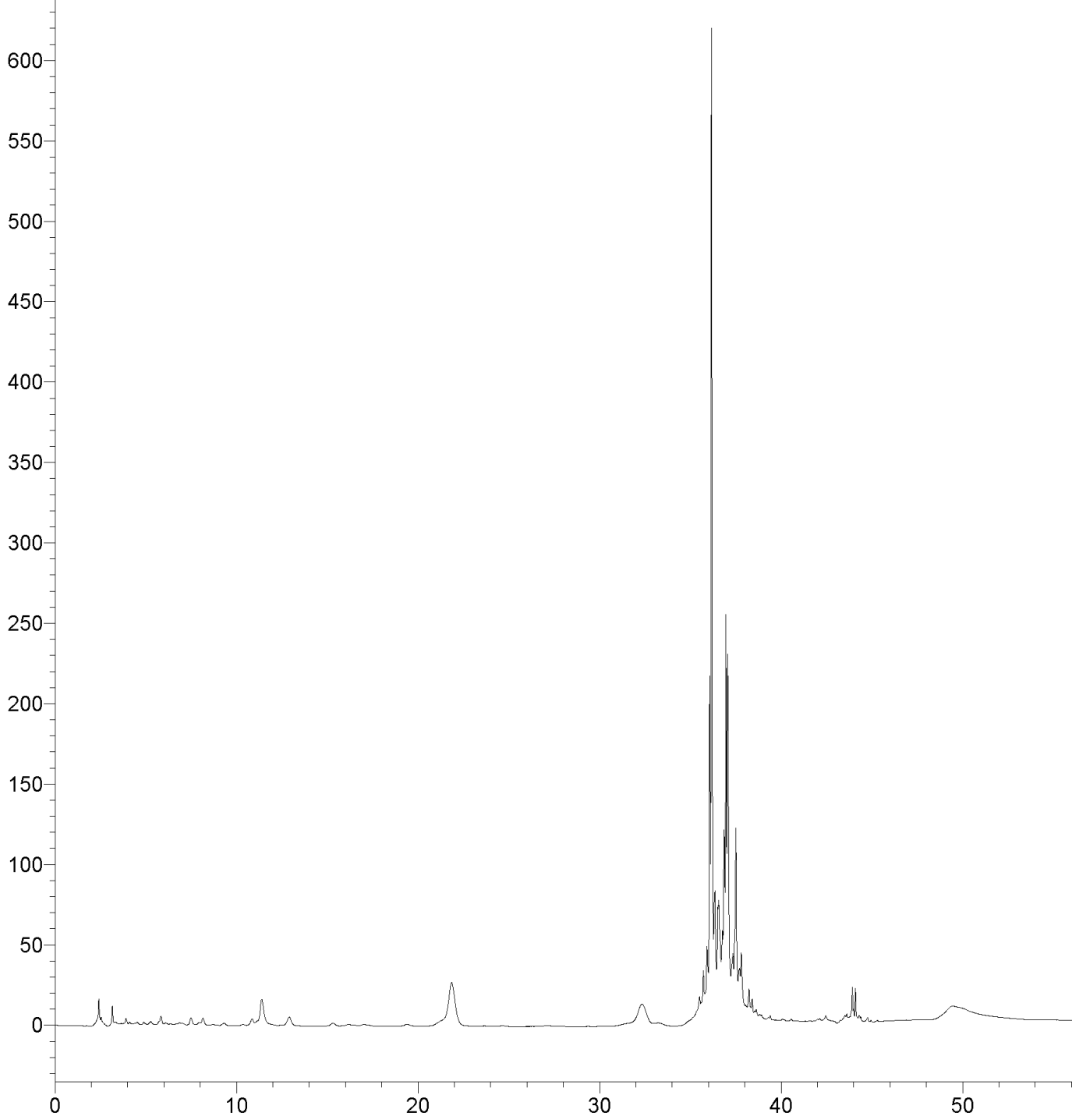


4

Time (Min)

Litter extract (spiked with Phloridzin)

Time (Min)

mAU


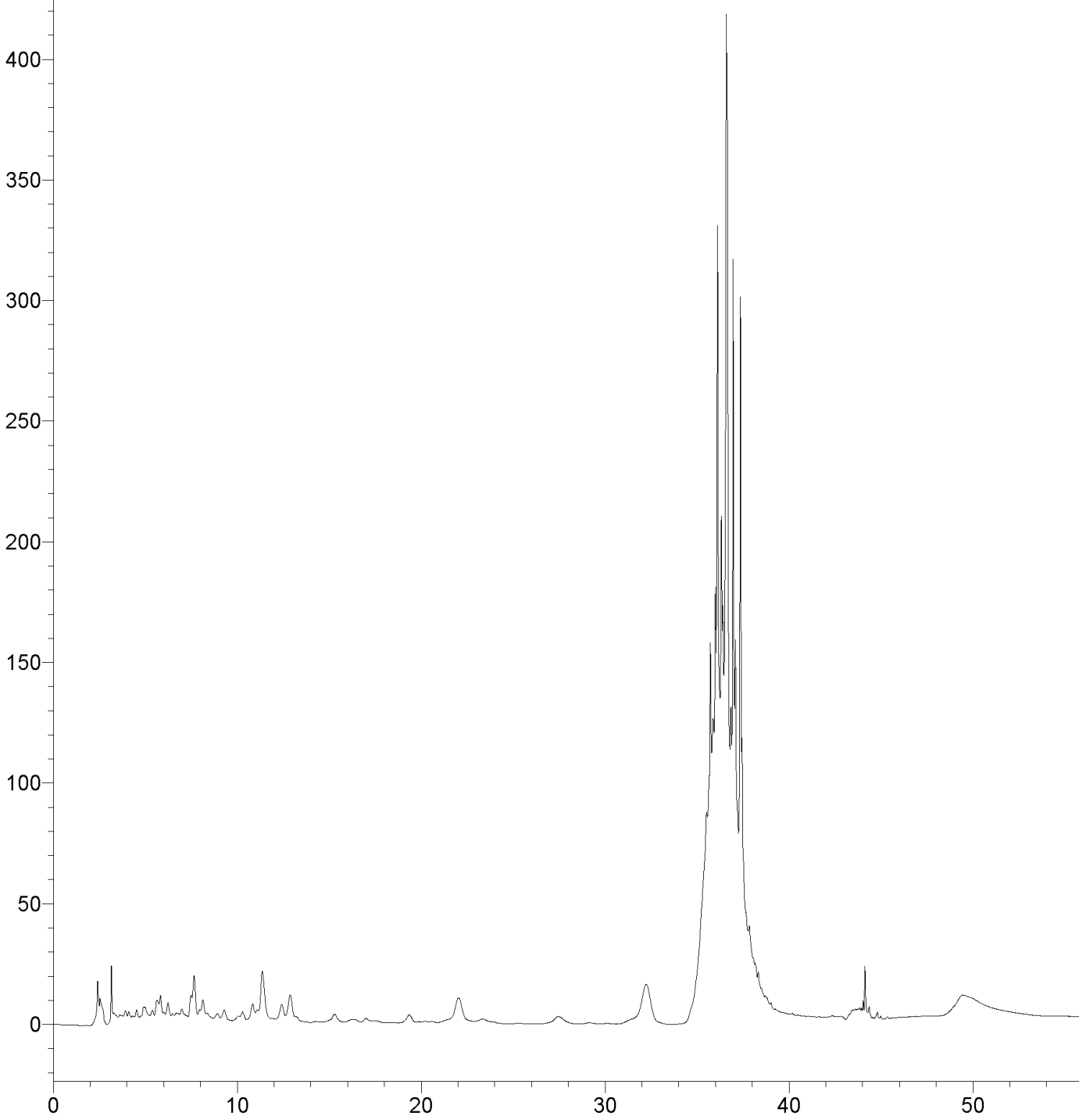


4
