# Supplementary figures and images for: Identification and Phytotoxicity Assessment of Phenolic Compounds in Chrysanthemoides monilifera subsp. monilifera (Boneseed)
Source: PLoS One. 2015 Oct 14;10(10):e0139992. doi: 10.1371/journal.pone.0139992 (PMC4605635; doi:10.1371/journal.pone.0139992)

S2 Fig.


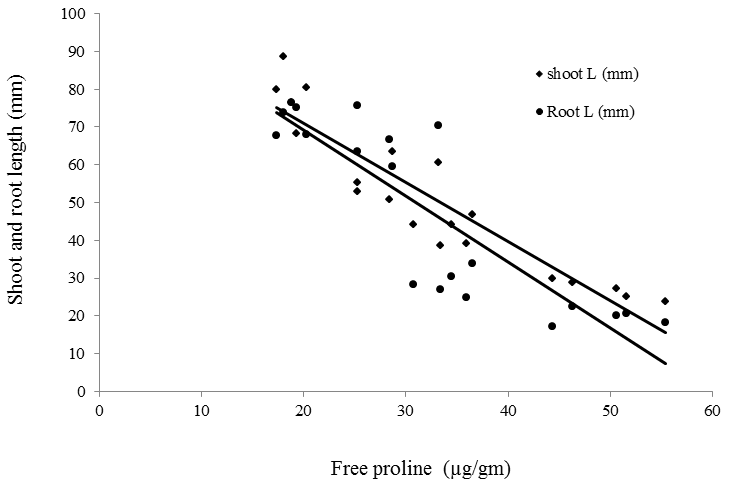


r= -0.92

r= -0.87

Supplement: S2 Fig — (DOCX) [file pone.0139992.s002.docx]
