# Supplementary material for: Identification and Phytotoxicity Assessment of Phenolic Compounds in Chrysanthemoides monilifera subsp. monilifera (Boneseed)
Source: PLoS One. 2015 Oct 14;10(10):e0139992. doi: 10.1371/journal.pone.0139992 (PMC4605635; doi:10.1371/journal.pone.0139992)
Supplement: S1 Table — (DOCX) [file pone.0139992.s003.docx]

S1 Table: Impact of standard phenolic compounds (dose response) on germination indices and biometric parameters of *I. axillaris* (Repeated experiment).

| Treatment | Parameters | | | | | | | |
| --- | --- | --- | --- | --- | --- | --- | --- | --- |
|  | TG | SpG | SpAG | CRG | HL | RL | HW | RW |
| Control | 67±2.7 | 2.19±0.08 | 12.71±0.45 | 7.97±0.04 | 3.83±0.12 | 8.28±0.28 | 0.40±0.02 | 0.25±0.01 |
| Catechin (µg/ mL) |  |  |  |  |  |  |  |  |
| 5 | 68±2.1 | 2.24±0.09 | 13.08±0.55 | 7.99±0.03 | 4.11±0.09 | 8.91±0.16 | 0.43±0.02 | 0.27±0.01 |
| 20 | 71±2.9 | 2.29±0.10 | 13.21±0.66 | 7.93±0.07 | 4.16±0.08 | 9.01±0.05 | 0.43±0.02 | 0.27±0.01 |
| 40 | 64±3.2 | 2.11±0.12 | 12.33±0.70 | 7.96±0.03^a^ | 3.87±0.09 | 7.29±0.15 | 0.37±0.01 | 0.21±0.01 |
| 80 | 63±1.4 | 2.03±0.05 | 11.69±0.29 ^a^ | 7.89±0.02^a^ | 3.20±0.09 | 5.97±0.14 | 0.30±0.02 | 0.17±0.01 |
| P-coumaric acid (µg/ mL) |  |  |  |  |  |  |  |  |
| 2.5 | 66±2.9 | 2.21±0.10 | 13.06±0.62 | 8.03±0.04 | 3.84±0.04 | 8.56±0.23^a^ | 0.39±0.01 | 0.24±0.01 |
| 10 | 68±4.0 | 2.26±0.14 | 13.09±0.81 | 7.98±0.03 | 3.98±0.09 | 9.65±0.17 | 0.41±0.02 | 0.28±0.02 |
| 20 | 72±4.0 | 2.33±0.15 | 13.50±0.90 | 7.94±0.04 | 4.02±0.12 | 8.78±0.15 | 0.42±0.02 | 0.24±0.01 |
| 40 | 69±4.6 | 2.27±0.15 | 13.11±0.84 | 7.97±0.03^a^ | 3.72±0.06 | 7.93±0.23 | 0.37±0.02 | 0.22±0.01 |
| Ferulic acid (µg/ mL) |  |  |  |  |  |  |  |  |
| 27.5 | 68±4.2 | 2.24±0.17 | 12.97±1.06 | 7.95±0.05 | 3.91±0.12 | 8.95±0.26 | 0.41±0.02 | 0.25±0.02 |
| 110 | 65±3.2 | 2.09±0.11 | 12.00±0.66 | 7.90±0.03^a^ | 3.32±0.09 | 7.17±0.12 | 0.33±0.02 | 0.20±0.01 |
| 220 | 61±2.9 | 1.66±0.08 | 8.20±0.44 | 7.45±0.02^a^ | 2.11±0.08 | 2.03±0.07 | 0.22±0.01 | 0.11±0.01 |
| 440 | 00 | 00 | 00 | 00 | 00 | 00 | 00 | 00 |
| Phloridzin (µg/ mL) |  |  |  |  |  |  |  |  |
| 16.25 | 67±4.1 | 2.17±0.15 | 12.53±0.87 | 7.95±0.02 | 4.11±0.11 | 8.83±0.13 | 0.44±0.02 | 0.25±0.01 |
| 65 | 68±4.4 | 2.15±0.14 | 12.22±0.73 | 7.89±0.02 | 4.01±0.12 | 8.34±0.19 | 0.41±0.02 | 0.23±0.01 |
| 130 | 65±3.5 | 2.08±0.14 | 11.88±0.88 | 7.90±0.04 | 3.60±0.09 | 7.78±0.14 | 0.36±0.01 | 0.21±0.01 |
| 260 | 64±2.9 | 1.95±0.11 | 10.58±0.76 | 7.79±0.06 | 2.87±0.15 | 6.88±0.22 | 0.29±0.02 | 0.19±0.01 |
| Mixture |  |  |  |  |  |  |  |  |
| 1 | 69±4.2 | 2.27±0.16 | 13.08±1.02 | 7.97±0.04 | 4.19±0.09 | 9.04±0.28^a^ | 0.45±0.02 | 0.26±0.01 |
| 2 | 62±4.0 | 1.93±0.14 | 10.84±0.80 | 7.85±0.03^a^ | 3.84±0.08 | 6.64±0.19 | 0.33±0.02 | 0.18±0.01 |
| 3 | 30±2.7 | 0.79±0.08 | 3.68±0.40^a^ | 7.38±0.05^a^ | 1.51±0.06 | 1.04±0.03 | 0.18±0.01 | 0.09±0.01 |
| 4 | 00 | 00 | 00 | 00 | 00 | 00 | 00 | 00 |

Data presented as average ± SE. TG= Total germination, SpG= Speed of germination, SpAG= Speed of accumulated germination, CRG= Coefficient of rate of germination, SL= Shoot length, RL= root length, SW= Shoot weight, RW= root weight.

^a^ significantly varied when compared with the data of first trial.
