# Supplementary material for: Identification and Phytotoxicity Assessment of Phenolic Compounds in Chrysanthemoides monilifera subsp. monilifera (Boneseed)
Source: PLoS One. 2015 Oct 14;10(10):e0139992. doi: 10.1371/journal.pone.0139992 (PMC4605635; doi:10.1371/journal.pone.0139992)
Supplement: S2 Table — (DOCX) [file pone.0139992.s004.docx]

S2 Table

| **Multiple Comparisons** | | | | | | | |
| --- | --- | --- | --- | --- | --- | --- | --- |
| Dunnett t (2-sided)^a^ | | | | | | | |
| Dependent Variable | (I) Treatment | (J) Treatment | Mean Difference (I-J) | Std. Error | Sig. | 95% Confidence Interval | |
|  |  |  |  |  |  | Lower Bound | Upper Bound |
| Total Germination (%) | Litter leachate | Control | -18.666667^*^ | 4.745368 | .014 | -32.25147 | -5.08186 |
|  | Soil leachate | Control | -2.666667 | 4.745368 | .804 | -16.25147 | 10.91814 |
| Speed of Germination | Litter leachate | Control | -.720844^*^ | .139171 | .004 | -1.11926 | -.32243 |
|  | Soil leachate | Control | -.122234 | .139171 | .611 | -.52065 | .27618 |
| Speed of Acc Germ | Litter leachate | Control | -4.857459^*^ | .894302 | .003 | -7.41762 | -2.29730 |
|  | Soil leachate | Control | -.843634 | .894302 | .571 | -3.40380 | 1.71653 |
| Coeff of Rate of Germ | Litter leachate | Control | -.261989^*^ | .057824 | .007 | -.42752 | -.09645 |
|  | Soil leachate | Control | -.049506 | .057824 | .624 | -.21504 | .11603 |
| Hypocotyl Length | Litter leachate | Control | -.700000^*^ | .143630 | .005 | -1.11118 | -.28882 |
|  | Soil leachate | Control | .113333 | .143630 | .665 | -.29784 | .52451 |
| Radical Length | Litter leachate | Control | -5.766667^*^ | .529654 | .000 | -7.28293 | -4.25040 |
|  | Soil leachate | Control | -.146667 | .529654 | .946 | -1.66293 | 1.36960 |
| Hypocotyl weight | Litter leachate | Control | -.093333 | .032886 | .052 | -.18748 | .00081 |
|  | Soil leachate | Control | .060000 | .032886 | .196 | -.03414 | .15414 |
| Radical Weight | Litter leachate | Control | -.710000^*^ | .080921 | .000 | -.94166 | -.47834 |
|  | Soil leachate | Control | .003333 | .080921 | .999 | -.22832 | .23499 |
| *. The mean difference is significant at the 0.05 level. | | | | | | | |
| a. Dunnett t-tests treat one group as a control, and compare all other groups against it. | | | | | | | |
