# Supplementary material for: Identification and Phytotoxicity Assessment of Phenolic Compounds in Chrysanthemoides monilifera subsp. monilifera (Boneseed)
Source: PLoS One. 2015 Oct 14;10(10):e0139992. doi: 10.1371/journal.pone.0139992 (PMC4605635; doi:10.1371/journal.pone.0139992)
Supplement: S3 Table — (DOCX) [file pone.0139992.s005.docx]

S3 Table

| Multiple Comparisons | | | | | | | |
| --- | --- | --- | --- | --- | --- | --- | --- |
| Dunnett t (2-sided)^a^ | | | | | | | |
| Dependent Variable | (I) Treatment | (J) Treatment | Mean Difference (I-J) | Std. Error | Sig. | 95% Confidence Interval | |
|  |  |  |  |  |  | Lower Bound | Upper Bound |
| Shoot length | 0.5 g L | Control | -22.14000^*^ | 3.18550 | .000 | -30.3979 | -13.8821 |
|  | 1 g L | Control | -36.20000^*^ | 3.18550 | .000 | -44.4579 | -27.9421 |
|  | 2 g L | Control | -51.80000^*^ | 3.18550 | .000 | -60.0579 | -43.5421 |
| Root length | 0.5 g L | Control | -5.06000 | 2.67414 | .180 | -11.9923 | 1.8723 |
|  | 1 g L | Control | -43.30000^*^ | 2.67414 | .000 | -50.2323 | -36.3677 |
|  | 2 g L | Control | -52.48000^*^ | 2.67414 | .000 | -59.4123 | -45.5477 |
| Shoot dry weight | 0.5 g L | Control | -12.89400^*^ | 1.39004 | .000 | -16.4975 | -9.2905 |
|  | 1 g L | Control | -20.88600^*^ | 1.39004 | .000 | -24.4895 | -17.2825 |
|  | 2 g L | Control | -25.02200^*^ | 1.39004 | .000 | -28.6255 | -21.4185 |
| Root dry weight | 0.5 g L | Control | -4.56000^*^ | .47761 | .000 | -5.7981 | -3.3219 |
|  | 1 g L | Control | -9.14600^*^ | .47761 | .000 | -10.3841 | -7.9079 |
|  | 2 g L | Control | -11.44800^*^ | .47761 | .000 | -12.6861 | -10.2099 |
| Leaf no | 0.5 g L | Control | -1.40000^*^ | .34278 | .002 | -2.2886 | -.5114 |
|  | 1 g L | Control | -2.60000^*^ | .34278 | .000 | -3.4886 | -1.7114 |
|  | 2 g L | Control | -4.00000^*^ | .34278 | .000 | -4.8886 | -3.1114 |
| Relative water content | 0.5 g L | Control | -1.50800^*^ | .49712 | .021 | -2.7967 | -.2193 |
|  | 1 g L | Control | -3.19800^*^ | .49712 | .000 | -4.4867 | -1.9093 |
|  | 2 g L | Control | -4.15800^*^ | .49712 | .000 | -5.4467 | -2.8693 |
| chlorophyll a | 0.5 g L | Control | -.33600^*^ | .03739 | .000 | -.4329 | -.2391 |
|  | 1 g L | Control | -.44200^*^ | .03739 | .000 | -.5389 | -.3451 |
|  | 2 g L | Control | -.51400^*^ | .03739 | .000 | -.6109 | -.4171 |
| Chlorophyll b | 0.5 g L | Control | -.12000^*^ | .02022 | .000 | -.1724 | -.0676 |
|  | 1 g L | Control | -.18000^*^ | .02022 | .000 | -.2324 | -.1276 |
|  | 2 g L | Control | -.25400^*^ | .02022 | .000 | -.3064 | -.2016 |
| Total chlorophyll | 0.5 g L | Control | -.45800^*^ | .05394 | .000 | -.5978 | -.3182 |
|  | 1 g L | Control | -.62200^*^ | .05394 | .000 | -.7618 | -.4822 |
|  | 2 g L | Control | -.76800^*^ | .05394 | .000 | -.9078 | -.6282 |
| Free proline | 0.5 g L | Control | 9.37400^*^ | 1.91112 | .000 | 4.4197 | 14.3283 |
|  | 1 g L | Control | 15.43600^*^ | 1.91112 | .000 | 10.4817 | 20.3903 |
|  | 2 g L | Control | 30.87400^*^ | 1.91112 | .000 | 25.9197 | 35.8283 |
| *. The mean difference is significant at the 0.05 level. | | | | | | | |
| a. Dunnett t-tests treat one group as a control, and compare all other groups against it. | | | | | | | |
